# Construction and validation of a video on the insertion of gastric and enteral tubes in children*

**DOI:** 10.1590/1518-8345.7226.4506

**Published:** 2025-03-31

**Authors:** Gabriela Beatriz Leonhardt, Giovani Basso da Silva, Guilherme Kayser Prates, Simone Travi Canabarro, Luccas Melo de Souza

**Affiliations:** 1 Santa Casa de Misericórdia de Porto Alegre, Hospital da Criança Santo Antônio, Porto Alegre, RS, Brazil.; 2 Scholarship holder at the Fundação de Amparo à Pesquisa do Rio Grande do Sul (FAPERGS), Brazil.; 3 Santa Casa de Misericórdia de Porto Alegre, Hospital Santa Rita, Porto Alegre, RS, Brazil.; 4 Universidade Federal de Ciências da Saúde de Porto Alegre, Porto Alegre, RS, Brazil.; 5 Universidade Federal de Ciências da Saúde de Porto Alegre, Departamento de Enfermagem, Porto Alegre, RS, Brazil.

**Keywords:** Enteral Nutrition, Child Health, Nursing, Nursing Education, Nursing Methodology Research, Instructional Film and Video

## Abstract

to construct and validate an educational video on the procedure for inserting gastric and enteral tubes in children.

a methodological study in three phases: a) pre-production of the video; b) production; and c) post-production. First, a script was created based on scientific literature. The video was recorded in the Simulation Laboratory and edited using Movavi^®^ software. The script and video were evaluated by 23 and 12 experts, respectively, via Google Forms^®^. The Health Education Content Validation, Video Validation, and Content Validity Index instruments were used, with a cut-off point ≥ 0.80.

the experts covered three regions of the country. The video contains scenes recorded with a pediatric simulation mannequin, images of materials used in the procedure, and screens with theoretical content. The video script obtained an overall content validation index of 0.83 and the video 0.94, both in one round. The final video lasts 10 minutes and 10 seconds.

the script and video were validated. It is freely available on YouTube^®^ and can be used by nurses/nursing students as a tool for study and professional assistance.

## Highlights

**(1)** Educational nursing videos require a scientific methodology to be produced. **(2)** The video script and the video were validated by the experts. **(3)** The material was rated as excellent and of excellent quality. **(4)** The video is freely accessible and can contribute to qualified and safe practices.

## Introduction

Gastric and enteral probing are routine procedures performed by nurses, especially in hospital settings, on patients of all ages^(1- 2)^. It consists of inserting a tube through the nostril or mouth into the stomach (nasogastric or orogastric tube) or intestine (nasoenteric or oroenteric tube). There is little epidemiological data on the use of these tubes, especially in children. In 2016, a multicenter study identified the use of gastric or enteral tubes in 24% of the 8,333 pediatric/neonatal patients admitted to 63 hospitals in the United States of America. There were 1,316 nasogastric tubes (66%), 414 orogastric tubes (21%) and 261 enteral tubes (17%)^(3)^.

In child care, gastric and enteral tubes are commonly used for nutritional intake when the oral route is not possible. Their purpose/indication is to administer diet and medication, hydration, as well as allowing for washing, drainage of liquids or air and collection of gastric material^(4- 5)^. Contraindications include intestinal obstructions, gastrointestinal perforations and certain congenital anomalies that make intervention impossible^(1, 5)^. The choice of placement of the tube, whether gastric or enteral, depends on the patient’s clinical condition and the specific need for therapy, with considerations about the duration of use, the risk of aspiration and the need for continuous feeding^(1, 6)^.

Inappropriate insertion or use of these devices can lead to adverse events such as nasopharyngeal discomfort, vomiting, epistaxis, erosion of the nasal septum, pressure injuries related to fixation, respiratory complications, and even death^(1, 5)^. A study of 130 nurses in Saudi Arabia found that more than half had unsatisfactory knowledge (53.1%) or incompetent practices (58.5%) when inserting gastric or enteral tubes^(7)^.

It is therefore essential that the nursing team’s actions aim to improve care processes and child safety, based on the best care practices. To this end, Permanent Health Education (PHE) for professionals is an essential strategy for improving care for children who need these tubes, as it promotes the constant updating of knowledge and avoids the obsolescence of practices in a scenario of rapid technological and scientific evolution^(8- 9)^.

Among these technologies, educational videos have been widely used in nursing education in recent years, as a strategy for quickly disseminating information on content/procedures. It makes it possible to synthesize and visualize theory and associate it with practice, adding dynamism to the content, enhancing the construction of knowledge, understanding and reflection, as long as it is properly developed, i.e. with methodological rigor^(8- 9)^.

Given the complexity of inserting gastric and enteral tubes, the constant evolution of scientific knowledge and care practices, as well as the differences in how the procedure is carried out^(7)^, it was considered important to create an educational video to guide the standardization of the stages of this procedure, in order to fill gaps in the continuing education of health professionals. This type of educational resource can help in the teaching-learning process and improve health care, depending on the method and theoretical framework used^(8)^. This study aimed to develop and validate an educational video on the procedure for inserting gastric and enteral tubes in children.

## Method

### Study design

This is a methodological study, with the production of an educational technology in video format. It was structured following the recommendations of the Revised Standards for Quality Improvement Reporting Excellence (SQUIRE 2) guide.

We used Fleming, Reynolds, and Wallace’s guide to making videos^(10)^, one of the most widely used methods for this type of production in nursing^(9)^. It was carried out in three phases: a) pre-production of the video; b) production and c) post-production. In phase 1, the video script was constructed and validated by experts. Phase 2 involved recording the video. In phase 3, the video was validated by the experts, final adjustments were made and it was made available.

Phase 1, pre-production of the video, was carried out in four stages, literature search, construction, validation, and updating of the script, to develop the production script. As there is no consensus or guidelines on the insertion technique and to identify the best practices for inserting gastric/enteral tubes in pediatrics, the search for theoretical content to build the script took place through a narrative review of the literature in the following databases: Cumulative Index to Nursing and Allied Health Literature (CINAHL), Medical Literature Analysis and Retrieval System Online (MEDLINE), SciVerse Scopus (SCOPUS) and Web of Science. Grey literature was used, with a search in the Theses and Dissertations catalog of the Coordination for the Improvement of Higher Education Personnel (CAPES) and in reference books in the area of Pediatrics.

After selecting the relevant information to compose the material, the content of the script was organized and made available on a board. Each row corresponded to a scene from the video and the three columns contained the following information: scene, description of the content of the scene, and technical description of the scene^(11)^. The script was drawn up by two PhD nurses and a nursing student, all authors of the study.

In the “description of the scene content” column, the actions carried out by the nurse, the technique for carrying out the procedure, the texts or speeches were specified. The “technical description of the scene” dealt with visual aspects, such as images, audio and/or video, based on a pre-existing study^(11)^. The script was prepared in 42 days.

In phase 2, video production, after modifying the script, the scenes and narrations described in pre-production were recorded. Complementary materials, such us articles, documents, among others, were selected to make up the product and screens with additional information were created using Canva^®^ software. Movavi^®^ software was used to edit the video.

Adjustments were made to the recordings of the scenes, as well as including the recorded scenes, narration and information screens in a single product. This phase took place between June 30, 2022 and March 17, 2023 (260 days), with three researchers recording and two editing. The entire team, two supervising professors and three undergraduate students, took part in the initial analysis of the video.

### Study site

The script (Phase 1) and video (Phase 2) were developed in the teaching laboratories of the Federal University of Health Sciences of Porto Alegre (UFCSPA), in Porto Alegre, Rio Grande do Sul, Brazil, with teachers and undergraduate students from the Bachelor of Nursing course. This university has 16 undergraduate courses, all linked to the health area. The materials were validated in a virtual environment, using the Lattes^®^ Platform and the Google Drive^®^ and Google Forms^®^ tools.

### Length

The study was carried out between March 2022 and August 2023.

### Population

To validate the script and video, experts were recruited from all over Brazil via the Lattes^®^ Platform, which openly gathers researchers’ CVs (“Lattes Curriculum”) and information on research groups and institutions in the country.

### Selection criteria

In phase 1, to validate the video script, experts were recruited in two ways. The first was through a search on the Lattes^®^ Platform, using the following keywords and Boolean operators: nursing AND gastrointestinal intubation AND pediatrics OR child health; nursing AND enteral nutrition AND pediatrics OR child health. The experts who responded to the data collection instrument were asked to nominate up to three other researchers to take part in the validation of the material by snowball technique. 101 invitations were sent out that is 66 via the Lattes^®^ Platform and 35 via the snowball technique.

The inclusion criteria for the experts were: being a nurse, working in Brazil and achieving at least five points in the adapted Fehring Criteria^(12)^, considering: a master’s degree and/or doctorate in Nursing (four points); article published in the area of interest (three points); training (specialization or course) in the area of interest (two points); professional practice (care, teaching or research) of two years or more in the area of interest; dissertation/thesis in the area of interest (one point). The area of interest was defined as “child health/pediatrics”. The exclusion criterion was: not having Brazilian Portuguese as their mother tongue, as this was the language of the video.

### Sample definition

The sample size was set at a minimum of six and a maximum of 20 experts, which was considered sufficient^(9)^. Once the emails had been sent and the 20 experts had been reached, another three completed the evaluation, finalizing the sample for the script evaluation with 23 experts nurses.

### Study variables

With regard to the experts, the following variables were collected: age, gender, city/state of work, length of time working in nursing, professional training/titles, area of expertise and current place of work. In relation to the validation of the script and video, the variables involved aspects related to the objects of study, validation of the script and video, collected using specific instruments.

### Instruments used for data collection

The data collection instruments were entered into Google Forms^®^. To evaluate the script, we used the Health Education Content Validation Instrument (HECVI)^(13)^, which contains 18 questions organized into three domains: objectives, structure/presentation, and relevance. The HECVI questions are in Likert scale format (0 = disagree, 1 = partially agree and 2 = totally agree). Eight questions were included to characterize the experts and one descriptive question for free-form criticisms and recommendations.

To validate the video, the Video Evaluation Instrument used in a previous study^(14)^ was adapted. It contains five dimensions that analyze functionality, usability, efficiency, audiovisual technique and the environment. This instrument also has a Likert scale, with three response options (0 = disagree, 1 = partially agree and 2 = totally agree) for the 11 items evaluated.

### Data collection

Validation of the video script - phase 1 - pre-production, was carried out in a virtual environment, using the tools available on the Google Drive^®^ platform, where the prepared script was stored in a non-editable text format. This stage, which took place between April 04, 2022 and May 20, 2022 allowed for the prior assessment of the experts, who analyzed the content of the script. HECVI^(13)^ and the instrument for characterizing the experts were used.

Once the experts had been identified, a message was sent, by e-mail, via the Lattes^®^ Platform, inviting them to take part. The message contained the e-mail addresses of the Free and Informed Consent Term (FICT), the video script, and the data collection instrument. Access was via Google Forms^®^. For those indicated by the snowball technique, an e-mail was sent with the same accesses.

Invitations were sent out every two weeks, except to those who said they were not interested in contributing to the study or had already responded. In the end, 23 experts responded to the invitations, making up the committee of experts. There were no exclusions among those who responded.

Based on the results of the script evaluation, the video script was updated (within 15 days). To do this, the experts’ answers to the qualitative (open-ended) question in the data collection instrument were taken into account. The answers were compared with the literature and, when convergent, included in the script.

The validation of the video - phase 3 - post-production of the video, was also carried out in a virtual environment and the 23 experts who made up the first phase committee were contacted. Each of them received five invitation emails with a link to Google Forms^®^ containing: a new FICT; the video link, available in a non-editable format; and the Video Validation Tool^(14)^. The video evaluation committee was made up of 12 experts. Video validation data collection took place between April 13, 2023, and June 6, 2023 (54 days).

After implementing the suggestions, made between June and August 2023, made by the experts, the video on gastric/enteral probing in Pediatrics was made available for free public access on the YouTube^®^ channel of the UFCSPA Research Group on Technologies, Management, Education, and Safety at Work (TeGEST).

### Data processing and analysis

The analysis took place on Google Sheets^®^ to validate the script and the video. The Content Validity Index (CVI) was applied and the domains and the overall assessment of the HECVI were considered valid when ≥ 0.8. The following calculation was used for the CVI: sum of answers 2 / sum of all answers^(15)^.

Descriptive statistics, mean, frequency, standard deviation, and mode, were used to characterize the experts. For the analysis and presentation of the answers to the open questions, they were identified by the letter “E”, plus their order number in the database, for example: “E1”, “E2”, [...], “E23”.

In phase 3, the post-production of the video, the cut-off point of 0.80 was also adopted and the same calculation was made for the CVI, when using the Video Validation Tool. The same system as in phase 1 was used in phase 3 to analyze the open questions.

### Ethical aspects

This study was approved by the Research Ethics Committee (REC) of UFCSPA, No. 5.039.912, and the FICT was used in the two data collections with the experts.

## Results

The study resulted in two products: a script and a video on the gastric/enteral tube procedure in pediatrics, aimed at nurses and nursing students.

Of the 23 nurses who took part in the study, 21 (91.3%) were female. Their ages ranged from 34 to 68, with a mean of 45.8±6.3 years. Concerning the experts’ places of residence, three Brazilian regions were covered, with 10 (43.5%) experts from the South, 9 (39.1%) from the Southeast and 4 (17.4%) from the Northeast. The Midwest and North regions had no experts. A sample was obtained from seven states, predominantly Rio Grande do Sul with 9 (39.1%) experts, São Paulo with 6 (26%) and Rio de Janeiro with 3 (13%). Rio Grande do Norte had 2 (8.6%) experts and Bahia, Pernambuco, and Paraná had 1 (4.3%) expert each.

The length of professional experience as a nurse ranged from eight to 45 years, with a mean of 21.9±7.8 years. The maximum qualifications of the experts were 11 (47.8%) masters, 10 (43.5%) doctors and 2 (8.7%) post-doctors. As for the area of expertise, 14 (60.9%) predominated in child and adolescent health, followed by neonatology, 7 (30.4%) and critical care, 4 (17.4%). All had experience in pediatrics/child health, according to the CV analysis.

With regard to the type of work institution, 12 (52.2%) worked in health services and 11 (47.8%) in teaching in higher education. The 23 experts obtained a score, according to the Fehring Criteria, ranging from eight to 12, with a mean of 11 points and a mode and median of 12.

The script was constructed with 26 scenes in mind. They covered hand hygiene, patient identification, interaction/bonding with the family and child, the materials needed, positioning the child, measuring, lubricating, inserting and securing the tube, as well as tests to confirm positioning. Table 1 shows the experts’ assessment of the eighteen items that make up the HECVI.

**Table 1 -:** Content validation of the video script, according to HECVI* (n = 23). Porto Alegre, RS, Brazil, 2022-2023

**Variables**	**CVI** ^†^
**Domain 1 - Objectives**	0.802
Contemplates the proposed theme	0.820
Suitable for the teaching-learning process	0.900
Clarifies doubts about the topic	0.562
Provides reflection on the topic	0.850
Encourages behavior change	0.833
**Domain 2 - Structure/Presentation**	0.816
Language appropriate to the target audience	0.930
Language appropriate to the educational material	0.904
Interactive language that allows active involvement in the educational process	0.833
Correct information	0.625
Objective information	0.850
Clarifying information	0.625
Necessary information	0.722
Logical sequence	0.820
Current topic	0.878
Appropriate text size	0.878
**Domain 3 - Relevance**	0.953
Stimulates learning	0.954
Contributes to knowledge in the field	0.954
Sparks interest in the topic	0.952
**Global CVI** ^†^	0.838

*HECVI = Health Education Content Validation Instrument; ^†^CVI = Content Validity Index

In the dissertation field, the experts (E2, E18, and E21) suggested detailing child- and family-centered care, including family members as active participants with the patient, in order to make the procedure less traumatic. They also mentioned including therapeutic toys as a way of facilitating the patient’s cooperation during probe insertion (E2, E18, and E21).

Three experts (E1, E13 and E17) proposed addressing the issue of gastric/enteral tube insertion being the sole responsibility of the nurse, when considering the nursing team, and clarifying the nurse’s competencies when carrying out this procedure. Five experts (E1, E4, E14, E16, and E22) mentioned the specificities of the procedure for neonatal patients, pointing out the need to detail these aspects in the material or exclude this audience. Two experts (E4 and E17) suggested replacing the term “catheter” with “probe” because the first term is associated with intravenous therapy.

Two experts (E19 and E20) said that attention should be paid to marking and securing the probe, as only the marking on the pen goes out, and that the tape should be well secured to prevent it from being swallowed by the child. Fixation should follow the institutional standard, regardless of whether it is on the cheek or nose (E19 and E20). Another recommendation for inclusion was the use of a hydrocolloid sheet to protect the child’s skin from the probe (E8, E17, E19, and E22).

Regarding catheter positioning tests, some nurses (E2, E15, and E20) recommended that there are references that contraindicate the auscultation test with the stethoscope, and that radiography is the gold standard. As for the volume of air injected for the auscultation test, three experts (E1, E18, and E19) stressed the need to describe that there is a difference according to age group. When it comes to removing the guidewire or mandrel from the catheter, it was pointed out that this is done after the catheter has been inserted and before the patient goes for radiography (E2, E13, E18, and E20).

As positive contributions, the experts rated the material as “very didactic” (E2) and “well prepared” (E22). Other experts congratulated the choice of topic (E15) and considered the topic, content, and format of the script to be “pertinent” (E13). The script was also characterized as “exquisite and of excellent quality” (E21).

After evaluation and validation by the committee of experts, the script was updated according to their considerations. The video was recorded at the UFCSPA Skills Laboratory, with its initial version being 10’1”, called: “Gastric/enteral probing in Pediatrics”.

The video has scenes recorded with a pediatric simulation mannequin, images of materials used in the procedure, and screens with theoretical content. Its content ranges from the preparation of materials to specific techniques for inserting and checking the correct positioning of the probe and includes different visual resources in the form of screens to provide theoretical and legal support. Table 2 shows the experts’ evaluation of the 13 items that make up the video evaluation tool.

**Table 2 -:** Video validation, according to the Video Validation Instrument (n = 12). Porto Alegre, RS, Brazil, 2022-2023

**Variables**	CVI*
**Domain 1 - Functionality**	0.909
The video clearly and adequately presents the important aspects of the procedure	0.909
The video facilitates the teaching-learning process of the procedure	0.909
**Domain 2 - Usability**	0.896
The video makes it easy to learn the theoretical concepts used and the technique of the procedure	0.857
The video allows professional nurses to replicate the correct technique of the procedure in their professional practice	0.857
Logical sequence	0.956
Appropriate text size	0.909
**Domain 3 - Effectiveness**	0.933
The length of the video is adequate to learn the content	0.909
The video is attractive/holds the viewer’s attention	0.956
**Domain 4 - Audiovisual Technique**	0.985
The quality of the video image is suitable for observing the scenes	0.956
The tone and voice of the narrators are clear and appropriate	1.000
The vocabulary used in the video is efficient and understandable to the target audience	1.000
**Domain 5- Environment**	1.000
The environment used to record the scenes is considered suitable	1.000
The material used to carry out the procedure is correct	1.000
Global CVI*	**0.942**

*CVI = Content Validity Index

In the descriptive field, the experts (E1; E5; E7; E17, and E19) made suggestions as to when to remove the guidewire and how to fix and measure the probe in the video. In addition, they were instructed to include that the doctor is the one who should release the use of the probe for enteral nutrition (E17).

With regard to probe positioning tests, it was suggested that the technique for auscultating the epigastric region should be more detailed (E22); that the pH test has low accuracy because it is influenced by substances that the gastric juice comes into contact with (E17); and that although x-rays are the gold standard, they should be used with caution (E5). It was also pointed out that more than one technique should be used to confirm the correct positioning of the probe (E5 and E21).

One expert (E17) mentioned that the size of the probe used for children can be from 6 French upwards and that when choosing the size of the probe, the needs of the child to be supplied by the device should be considered (E5). It was recommended to list the aspects that differentiate gastric and enteral probing, the types of probes that exist, the contraindications for carrying out the procedure, and the guidance that should be given to the family about the procedure (E17 and E22).

Regarding the audiovisual technique, experts E7 and E19 pointed out the short display time of some of the information screens. They advised on how to narrate all the information in the material (E7); distribute the information contained in each screen over more screens (E1), and show the moment when the probe is fixed and the mandrel is removed from a closer angle (E19 and E21).

The suggestions made by the experts were analyzed and compared with the literature and then included. The video was updated and the final version contains lasts 10 minutes and 10 seconds. The video was then made available on the Research Group’s YouTube^®^ channel via the link https://bit.ly/47Suc7l.

## Discussion

The predominance of nurses in their middle adult years among the experts reflects data from a survey involving the profession in Brazil, in which 86.2% are women and 66.6% are over the age of 40^(16)^. The sample included experts from three of Brazil’s five regions, with no evaluators from the Midwest or North. This corroborates the data from the Brazilian survey, which shows a lower concentration of nurses in these two regions of the country, 8.2% and 6.7%, respectively^(16)^.

When analyzing the Federative Unit of the participants, the state of Rio Grande do Sul accounted for 39.1% of the sample. It is likely that the higher number of nurses from this state is justified by the origin of the study.

The length of professional experience and qualifications of the experts is similar to that of other studies that have validated educational materials, in which higher qualifications and expertise are required^(17- 22)^. The average Fehring score (adapted) was 11, ranging from eight to 12 points. Therefore, these results showed the different profiles of the participants in this study, thus validating the committee’s expertise.

In constructing the script/video, we sought to present theoretical content related to the gastric/enteral tube procedure, such as concept, indications/contraindications, objectives, legal support, necessary materials, and playful techniques. In addition, some controversial points were covered when carrying out the procedure, such as measuring the length and diameter of the probe, the position/restraint of the child, stabilizing the device, and positioning confirmation tests.

In the last five years, there has been a growth in the production of educational videos in nursing education, fuelled by the digital explosion, recent teaching demands, and changes in student profiles, with the insertion of new educational tools. An integrative review published in 2023 found 19 articles on the production of videos in nursing, most of which used the pre-production, production, and post-production phases, with validation by experts, as occurred in this investigation^(9)^.

This construction method has shown promising results when analyzing the outcome of the video in the target population. A Randomised Clinical Trial (RCT) that sought to assess the effectiveness of an educational video on the knowledge of 100 hospitalized patients about safe practices in the perioperative period identified a significantly higher gain in knowledge (t =3.72 ±1.84; p<0.001) in the intervention group (educational video) than in the control group (standard guidelines)^(23)^.

Another study showed that Portuguese-speaking nursing students acquired more knowledge about peripheral venipuncture after using a validated educational video on the subject^(24)^. Therefore, the proposal to construct and validate educational videos aimed at nursing care situations can favor the development of professionals’ knowledge and skills^(25- 26)^, as proposed in this study. When not evaluated by experts, the material offered can contain errors in content and have an unattractive design, which can discourage use by the target audience and encourage inappropriate practices^(27)^.

The script for the video on gastric/enteral probing in children achieved an overall CVI of 0.838 and the video an overall CVI of 0.942, with each domain ranging from 0.8002 to 0.952 (script) and 0.896 to 1.0 (video). Both products are therefore considered appropriate^(15)^. Other studies that validated educational videos, with different themes, populations, and instruments, also used a cut-off point of 0.8 for the CVI^(23, 25, 28- 30)^.

A methodological study that constructed and validated an educational video for nursing students on obstetric cardiopulmonary arrest obtained an overall CVI of 0.99, with the HECVI domains ranging from 0.95 to 1^(28)^. Another study, using the same instrument, validated seven educational videos to promote health and safety at work for professionals working in Primary Health Care, with the overall domain ranging from 0.88 to 0.96. In the “objectives” domain, the CVI ranged from 0.84 to 0.95; in “structure/presentation” from 0.91 to 0.99; and “relevance” from 0.81 to 0.95^(25)^ similar to this study.

The validation of the script added quality to the content for the subsequent production of the video, which had a higher CVI than the script. The contributions of the experts who evaluated the script and the video were essential for improving the information and guaranteeing the quality of the products produced, especially with regard to controversial content such as measuring the length of the probe and confirmatory tests for positioning. As the suggestions were specific, which was allowed by the open questions in the instruments, and all the CVI values in the domains of the two instruments were higher than the established cut-off point (0.80), one round of evaluation was maintained and the changes were made by analyzing the comments, comparing them with the literature^(31- 34)^ and the authors’ final decision.

When validating the video script, observations were made about the objectives of the procedure and the need to include the resolutions of the Federal Nursing Council (COFEN) on the gastric/enteral tube procedure and the mechanical restraint of the patient, which were added. The experts also pointed out the need to emphasize child- and family-centered care to make hospitalization and the procedures carried out during this period less traumatic. To provide comprehensive care for pediatric patients, the procedure must be clearly explained to the child, according to their stage of development, and to their family members, including them in the care. One way of doing this is by using a therapeutic toy, which is an attractive tool for the child and allows them to playfully demonstrate what is going to be done, making for a more peaceful and humanized procedure^(35)^.

At first, in the script, there was no distinction between the procedure and the different age groups in Pediatrics. Because of this, the experts stressed the importance of addressing the specificities of the neonatal period. This period is characterized from birth to the 28th day of life and is a restricted phase^(36)^. Therefore, when checking the particularities of this phase of development, it was decided not to include this audience in the material, since most of the target audience is concentrated after the 28th day of life. This decision was made clear in the final script and video.

Some experts mentioned the need to change the term “catheter”, initially used in the script, for the term “probe”, which is traditionally and historically used in health services and university environments. The decision was made to change the term “catheter” to “probe”, as this is the term most commonly used in the country, although technically the use of “catheter” or “tube” is appropriate, and the latter is the one most commonly used in English-language publications^(1- 2, 5- 7, 31- 33)^.

Once the gastric/enteral tube has been inserted, certain precautions must be taken, such as ensuring that the device is properly located in the gastrointestinal tract. In this regard, experts have stated that best practices no longer indicate the need for auscultation of the epigastric region, but rather the need for radiography. Some studies do not recommend and discourage the use of the auscultation test due to the possibility of auscultating noises in the epigastric region, regardless of whether the tip of the probe is located in the stomach, esophagus, or respiratory tract^(31)^. In line with the experts’ contributions and the findings in the literature^(31- 33)^, these two aspects were highlighted in the script when it came to the tests to confirm the positioning of the probe.

There is disagreement in the literature as to the accuracy of the methods. Because of this, the video emphasized the importance of always performing two bedside techniques to confirm the correct positioning of the probe before using it^(31- 33)^. X-ray is considered the gold standard for confirming the positioning of the probe, however, as mentioned by the evaluator and incorporated into the video, it should be used with caution, as repetitive exposure to radiation can be dangerous for the child^(31- 33)^.

The accuracy of the pH test was questioned by an expert, as pH can be influenced by the administration of a recent diet and the use of medication that inhibits gastric secretion. The literature shows that there is no significant difference between patients who have received medication or food and those who have not, but it can cause confusion for professionals, and it is important to use a second technique to confirm the position^(31- 33)^. Given this, the guidelines on the pH test in the video were maintained, as this can be considered a sensitive test for identifying the positioning of the probe^(31- 33)^.

One expert pointed out the need to better describe the auscultation test of the epigastric region. This technique was described in the video script but had not been incorporated into the video in text format, as it had been demonstrated in the filming. In order to clarify the information, a description of the technique was added to the video.

Another important precaution to ensure patient comfort concerns the skin and mucous membranes. Given this, some experts stressed the importance of using hydrocolloid sheets to pre-attach the probe. This feature was accepted in the corrections made to the script, as it is a technology that reduces humidity, friction, and shear, indicated to prevent pressure injuries, including those related to the use of medical devices^(37)^.

However, it is worth pointing out that, as it is an expensive material, it will not always be easy to access at the most different levels of care and/or services, especially considering the differences in health services in Brazil.

When validating the script and then the video, the experts discussed the different ways of fixing/stabilizing the probe, i.e. using string or stabilizing it in the cheek, chin, or temporomandibular region. Due to the different guidelines, what was already in the initial script was maintained, since there is no gold standard for how to do it and the peculiarities of the patient must be taken into account, as well as following the guidelines of the institution where the professional works, always taking into account the safety and comfort of the patient, while ensuring skin care. The script included an observation regarding the fixation of the probe when inserted orally, which had not been specified previously. In this study, it was considered that the probe should be fixed to the child’s cheek using microporous adhesive tape, and to the nose if placed through the nostrils^(38)^. When inserted orally, the probe should be positioned centrally and stabilized above the upper lip when possible^(39)^.

There is still a divergence of knowledge regarding how to insert and maintain the gastric/enteral probe, with some gaps in the literature. It is understood that technical-scientific rigor must be applied and that patient safety must be guaranteed at all stages of the procedure. An example of this divergence is the timing of the removal of the guidewire, pointed out by the experts who evaluated the script and the video, who made different recommendations. Some authors recommend removing it after the x-ray has been taken^(38)^, while others recommend removing it immediately after the catheter has been inserted and the device has stabilized on the skin, which can facilitate the migration of the probe tip and its final positioning even before the imaging exam^(40)^. In this study, we opted to adopt the recommendation to remove the guidewire after the positioning tests had been carried out and before the patient was referred for x-rays^(34, 40)^.

When validating the video, it was recommended that we review how to measure the length of the probe to be inserted. Various approaches are described in the literature, such as measurement based on the morphological aspects NEX (Nose, Earlobe, Xiphoid) and NEMU (Nose, Earlobe, Mid-Umbilicus) for gastric probing and the NEX method plus four centimeters for enteral probing^(31, 33)^. In addition, the experts highlighted the possibility of measuring based on age and height. It was decided to maintain the form used in the video, with the gastric position being measured from the tip of the nose/edge of the mouth to the earlobe, and from there to the middle space between the xiphoid appendix and the umbilical scar; and the enteral position from the tip of the nose to the earlobe, from there to the xiphoid appendix and then to the umbilical scar^(34)^.

The experts also made suggestions about the caliber of the probe, which were implemented in the video. The first refers to the fact that probes can be used from 6 French in children, as pointed out in the literature^(34)^, and in the video it was recommended from 6 French^(36)^. It should be noted that when choosing the probe size, the smallest diameter should be chosen, taking into account the characteristics of the child and the therapeutic needs to be met by the device.

Some suggested information was not added, such as contraindications for performing the procedure and the aspects that differentiate gastric and enteral probing, to avoid a long video. However, they are available as supplementary material to the video, accessed via the Quick Response Code and/or in the video description on YouTube^®^. Regarding the length (10 minutes and 10 seconds), it is believed that this influences the viewer’s involvement and learning, respecting the recommendations that stipulate six to 15 minutes^(41- 42)^, and similar to other studies that have validated educational videos for Nursing^(28- 30, 43- 44)^.

As for the audiovisual technique, the video’s authors opted to increase the display time of some of the informative screens, in order to allow reading without having to pause the video. In addition, the angle at which the probe was fixed and the mandrel removed was changed, as recommended. However, the suggestion that all the information in the material should be narrated was not accepted.

The script and video, prepared with scientific rigor and validated by experts, can be used as a strategy to diversify the teaching-learning processes of nurses and nursing students, in the classroom, in the laboratory, or complementary activities. The video entitled: “Gastric/enteral tube feeding in Pediatrics”, based on the script, can be used by the professional/student when performing the procedure, and is easy to consult, especially as it is available on freely accessible platforms on the Internet, such as YouTube®. The script can be accessed via the link provided in the description of the video, as well as the other materials that contributed to its development.

The results and the product (video) are original and unprecedented, as there is no published study on the production of an educational video on the target audience and the subject. As such, its originality and potential to help professionals develop theoretical and practical skills in the topic of gastric and enteral probing in children are highlighted.

A limitation is the greater local-regional participation of the experts who validated the products, which may limit the use of the products in the Central-West and Northern Regions of Brazil, due to the different cultures in the country. The loss of 11 experts in the last stage does not invalidate the results, since the 12 video evaluators exceeded the minimum stipulated in the literature, which is six^(15)^. As no other investigations were found involving the subject and the target audience (which exalts its originality), there is a restriction on comparing the data with other studies. Finally, the use of the CVI, which is fundamental in the product development process, is limited by the subjectivity of the experts^(15)^, which we sought to minimize with decisions based on the literature.

## Conclusion

The video “Gastric/enteral tube feeding in Pediatrics” was validated by the committee of experts. The study contributes to the advancement of scientific knowledge and nurses’ care practice by providing an educational resource that contains scientifically rigorous content, which is easily accessible and free of charge.

The product can be used independently in different educational proposals, as long as the Creative Commons Attribution 4.0 International license is complied with. Further studies are suggested to analyze its effect on the knowledge and skills of nurses and academics.
