# Supplementary material for: Construction and validation of a video on the insertion of gastric and enteral tubes in children
Source: Rev Lat Am Enfermagem. 2025 Mar 31;33:e4506. doi: 10.1590/1518-8345.7226.4506 (PMC11960621; doi:10.1590/1518-8345.7226.4506)
Supplement: Translation

# Translation: Construção e validação de vídeo sobre a inserção de sondas gástrica e enteral em crianças*

**DOI:** 10.1590/1518-8345.7226.4507

**Authors:** Gabriela Beatriz Leonhardt, Giovani Basso da Silva, Guilherme Kayser Prates, Simone Travi Canabarro, Luccas Melo de Souza

**Affiliations:** 1 Santa Casa de Misericórdia de Porto Alegre, Hospital da Criança Santo Antônio, Porto Alegre, RS, Brasil.; 2 Bolsista da Fundação de Amparo à Pesquisa do Rio Grande do Sul (FAPERGS), Brasil.; 3 Santa Casa de Misericórdia de Porto Alegre, Hospital Santa Rita, Porto Alegre, RS, Brasil.; 4 Universidade Federal de Ciências da Saúde de Porto Alegre, Porto Alegre, RS, Brasil.; 5 Universidade Federal de Ciências da Saúde de Porto Alegre, Departamento de Enfermagem, Porto Alegre, RS, Brasil.

**Keywords:** Nutrição Enteral, Saúde da Criança, Enfermagem, Educação em Enfermagem, Pesquisa Metodológica em Enfermagem, Filme e Vídeo Educativo

## Abstract

construir e validar um vídeo educacional sobre o procedimento de inserção das sondas gástrica e enteral em crianças.

estudo metodológico em três fases: a) pré-produção do vídeo; b) produção; e c) pós-produção. Primeiro, criou-se um roteiro embasado na literatura científica. O vídeo foi gravado em Laboratório de Simulação e editado no *softwareMovavi* ^®^. O roteiro e o vídeo foram avaliados por 23 e 12 especialistas*,* respectivamente, via *Google Forms* ^®^. Utilizaram-se os instrumentos de Validação de Conteúdo Educativo em Saúde, de Validação de Vídeo e o Índice de Validade de Conteúdo, com ponto de corte ≥ 0,80.

os especialistas contemplaram três regiões do país. O vídeo contém cenas gravadas com manequim de simulação pediátrica, imagens de materiais usados no procedimento e telas com conteúdo teórico. O roteiro do vídeo obteve índice de validação de conteúdo global de 0,83 e o vídeo de 0,94, ambos em uma rodada. O vídeo final tem duração de 10 minutos e 10 segundos.

o roteiro e o vídeo foram validados. Ele está disponível gratuitamente no *YouTube* ^®^ e pode ser utilizado por enfermeiros/acadêmicos de Enfermagem como um instrumento para o ensino e a assistência profissional.

## Destaques

**(1)** Vídeos educativos de Enfermagem requerem metodologia científica para sua elaboração. **(2)** O roteiro do vídeo e o vídeo foram validados pelos *experts.* **(3)** O material foi classificado como primoroso e de excelente qualidade. **(4)** O vídeo é de livre acesso e pode contribuir para as práticas qualificadas e seguras.

## Introdução

A sondagem gástrica e enteral são procedimentos rotineiros exercidos pelo enfermeiro, sobretudo no contexto hospitalar, em pacientes de todas as idades^(1 - 2)^. Consiste na inserção de uma sonda (ou tubo) pela narina ou pela boca até o estômago (sondagem nasogástrica ou orogástrica) ou intestino (sondagem nasoentérica ou oroentérica). Dados epidemiológicos sobre o uso dessas sondas são escassos, especialmente em crianças. No ano de 2016, um estudo multicêntrico identificou o uso de sonda gástrica ou enteral em 24% dos 8.333 pacientes pediátricos/neonatais internados em 63 hospitais dos Estados Unidos da América. Havia 1.316 sondas nasogástricas (66%), 414 orogástricas (21%) e 261 enterais (17%)^(3)^.

No cuidado à criança, as sondas gástricas e enterais são comumente utilizadas para o aporte nutricional quando a via oral não é possível. Tem, como finalidade/indicação, administrar dieta e medicamentos, hidratação, além de permitir a lavagem, drenagem de líquidos ou de ar e a coleta de material gástrico^(4 - 5)^. Entre as contraindicações, destacam-se as obstruções intestinais, perfurações gastrointestinais e certas anomalias congênitas que impossibilitem a intervenção^(1 , 5)^. A escolha do posicionamento da sonda, se gástrica ou enteral, depende do quadro clínico do paciente e da necessidade específica da terapia, com considerações sobre a duração do uso, o risco de aspiração e a necessidade de alimentação contínua^(1 , 6)^.

A inserção ou o uso inadequado desses dispositivos podem ocasionar eventos adversos, como desconforto nasofaríngeo, vômito, epistaxe, erosão do septo nasal, lesões por pressão relacionadas à fixação, complicações respiratórias e até a morte^(1 , 5)^. Um estudo conduzido com 130 enfermeiros da Arábia Saudita apontou que mais da metade tinha conhecimento insatisfatório (53,1%) ou práticas incompetentes (58,5%) na inserção das sondas gástricas ou enterais^(7)^.

Assim, é fundamental que as ações da equipe de Enfermagem visem à qualificação dos processos assistenciais e à segurança da criança, fundamentadas nas melhores práticas assistenciais. Para isso, a Educação Permanente em Saúde (EPS) dos profissionais surge como uma estratégia essencial para aprimorar o cuidado às crianças que necessitam de sondas, já que promove a atualização constante dos conhecimentos e evita a obsolescência das práticas em um cenário de rápida evolução tecnológica e científica^(8 - 9)^.

Dentre as tecnologias, o vídeo educativo tem sido amplamente utilizado no ensino de Enfermagem nos últimos anos, sendo uma estratégia para a disseminação rápida das informações sobre os conteúdos/procedimentos. Ele possibilita sintetizar e visualizar a teoria e associá-la à prática, agregando dinamicidade ao conteúdo, potencializando a construção do conhecimento, a compreensão e a reflexão, desde que adequadamente desenvolvido, ou seja, com rigor metodológico^(8 - 9)^.

Frente a complexidade da inserção das sondas gástrica e enteral, a constante evolução do conhecimento científico e das práticas assistenciais, bem como as divergências na execução do procedimento^(7)^, considerou-se importante construir um vídeo educativo que oriente a padronização das etapas desse procedimento, visando preencher lacunas na formação contínua dos profissionais de saúde. Esse tipo de recurso educativo pode auxiliar no processo de ensino-aprendizagem e qualificar a assistência à saúde, a depender do método e do referencial teórico utilizado^(8)^. Dessa forma, este estudo objetivou construir e validar um vídeo educacional sobre o procedimento de inserção das sondas gástrica e enteral em crianças.

## Método

### Delineamento do estudo

Trata-se de um estudo metodológico, com a produção de uma tecnologia educacional em formato de vídeo. Para a sua estruturação, seguiram-se as recomendações do guia *Revised Standards for Quality Improvement Reporting Excellence* (SQUIRE 2).

Utilizou-se o guia de elaboração de vídeos de Fleming, Reynolds e Wallace^(10)^, um dos métodos mais utilizados para esse tipo de produção na Enfermagem^(9)^. O estudo foi conduzido em três fases: a) pré-produção do vídeo; b) produção e c) pós-produção. Na fase 1, ocorreu a construção do roteiro do vídeo e sua validação por especialistas. A fase 2 constituiu a gravação do vídeo. Na fase 3, o vídeo passou pela validação de especialistas, pelos ajustes finais e pela disponibilização.

A fase 1, de pré-produção do vídeo, foi realizada em quatro etapas: busca na literatura, construção, validação e atualização do tema, a fim de desenvolver o roteiro da produção. Como não há consenso ou *guidelines* sobre a técnica de inserção e objetivando-se identificar as melhores práticas na inserção de sonda gástrica/enteral em Pediatria, a procura de conteúdo teórico para a construção do roteiro ocorreu por revisão narrativa da literatura nas seguintes bases de dados: *Cumulative Index to Nursing and Allied Health Literature* (CINAHL), *Medical Literature Analysis and Retrieval System Online* (MEDLINE), *SciVerse Scopus* (SCOPUS) e *Web of Science*. Utilizou-se a literatura cinzenta, com busca no catálogo de Teses e Dissertações da Coordenação de Aperfeiçoamento de Pessoal de Nível Superior (CAPES) e em livros de referência da área da Pediatria.

Após selecionadas as informações relevantes para compor o material, o conteúdo do roteiro foi organizado e disponibilizado em um quadro. Cada linha correspondia a uma cena do vídeo e as três colunas continham as seguintes informações: cena, descrição do conteúdo da cena e descrição técnica da cena^(11)^. O roteiro foi elaborado por dois enfermeiros doutores e por uma acadêmica de Enfermagem, autores do estudo.

Na coluna “descrição do conteúdo da cena” foram especificadas as ações realizadas pela enfermeira, a técnica para a execução do procedimento, os textos ou falas. Na “descrição técnica da cena” foram abordados os aspectos visuais, como imagens, áudio e/ou vídeo, embasado em estudo pré-existente^(11)^. O roteiro foi elaborado em 42 dias.

Na fase 2, de produção do vídeo, após a modificação do roteiro, ocorreu a gravação das cenas e as narrações descritas na pré-produção. Foram selecionados os materiais complementares, tais como artigos, documentos, entre outros, para compor o produto e criar as telas com informações adicionais, produzidas no *softwareCanva* ^®^. Para editar o vídeo, utilizou-se o *softwareMovavi* ^®^.

Foram realizados os ajustes nas gravações das cenas, além de incluir, em único produto, as cenas gravadas, a narração e as telas informativas. Essa fase ocorreu entre 30 de junho de 2022 e 17 de março de 2023 (260 dias), contemplando três pesquisadores para a gravação e dois para a edição. Toda a equipe composta por dois professores orientadores e três alunos de graduação participaram da análise inicial do vídeo.

### Local do estudo

A construção do roteiro (Fase 1) e do vídeo (Fase 2) ocorreu nos laboratórios de ensino da Universidade Federal de Ciências da Saúde de Porto Alegre (UFCSPA), em Porto Alegre, no Rio Grande do Sul, Brasil, com professores e bolsistas de iniciação científica do curso de Bacharelado em Enfermagem. Tal universidade possui 16 cursos de graduação, todos vinculados à área da saúde. A validação dos materiais foi realizada em ambiente virtual, utilizando-se a Plataforma Lattes^®^ e as ferramentas do *Google Drive* ^®^ e *Google Forms* ^®^.

### Período

O estudo foi desenvolvido entre os meses de março de 2022 e agosto de 2023.

### População

Para a fase de validação do roteiro e do vídeo, especialistas foram recrutados em todo o território brasileiro, via Plataforma Lattes^®^, que reúne, de forma aberta, o currículo de pesquisadores (Currículo Lattes) e informações de grupos de pesquisa e instituições no país.

### Critérios de seleção

Na fase 1, para a validação do roteiro do vídeo, os especialistas foram recrutados de duas formas. Os primeiros por busca na Plataforma Lattes^®^, utilizando-se as seguintes palavras-chave e operadores booleanos: enfermagem and intubação gastrointestinal and pediatria or saúde da criança; enfermagem and nutrição enteral and pediatria or saúde da criança. Aos especialistas que responderam o instrumento de coleta de dados, foi solicitada a indicação de até outros três pesquisadores para participarem da validação do material pela técnica bola de neve. Foram enviados 101 convites, sendo 66 pela Plataforma Lattes^®^ e 35 via técnica bola de neve.

Os critérios de inclusão para os especialistas foram: ser enfermeiro, atuar no Brasil e alcançar, no mínimo, cinco pontos nos Critérios de Fehring^(12)^ adaptados, considerando: mestrado e/ou doutorado em Enfermagem (quatro pontos); artigo publicado na área de interesse (três pontos); capacitação (especialização ou curso) na área de interesse (dois pontos); prática profissional (assistencial ensino ou pesquisa) de dois anos ou mais na área de interesse; dissertação/tese na área de interesse (um ponto). A área de interesse foi definida como “saúde da criança/pediatria”. Como critério de exclusão, adotou-se: não possuir como língua materna o português do Brasil, por ser esse o idioma do vídeo.

### Definição da amostra

A amostra foi estipulada considerando o mínimo de seis e o máximo de 20 especialistas apontada como suficiente^(9)^. Após o término do envio de *e-mails* e o alcance dos 20 especialistas, outros três completaram a avaliação, finalizando a amostra para a avaliação do roteiro com 23 enfermeiros especialistas.

### Variáveis do estudo

Para a caracterização dos especialistas foram coletadas as seguintes variáveis: idade, sexo, cidade/estado de atuação, tempo de atuação na Enfermagem, formação/titulação profissional, área de especialização e atual local de atuação profissional. Em relação à validação do roteiro e do vídeo, as variáveis envolviam aspectos relacionados aos objetos de estudo, ou seja, validação do roteiro e do vídeo, coletadas, utilizando-se os instrumentos específicos.

### Instrumentos utilizados para a coleta de dados

Os instrumentos para a coleta de dados foram inseridos no *Google Forms* ^®^. Para a avaliação do roteiro, utilizou-se o Instrumento de Validação de Conteúdo Educativo em Saúde (IVCES)^(13)^, que contém 18 questões organizadas em três domínios: objetivos, estrutura/apresentação e relevância. As questões do IVCES são em formato de escala de Likert, com três opções de respostas (0 = discordo, 1 = concordo parcialmente e 2 = concordo totalmente). Foram incluídas oito questões para a caracterização dos especialistas e uma questão descritiva para as críticas e recomendações de forma livre.

Na validação do vídeo, foi adaptado o Instrumento de Avaliação de Vídeo utilizado em estudo prévio^(14)^. Ele contém cinco dimensões que analisam a funcionalidade, usabilidade, eficiência, técnica audiovisual e o ambiente. Esse instrumento também apresenta uma escala de Likert, com três opções de respostas (0 = discordo, 1 = concordo parcialmente e 2 = concordo totalmente) para os 11 itens avaliados.

### Coleta de dados

A validação do roteiro do vídeo denominada fase 1 – pré-produção foi realizada em ambiente virtual, utilizando as ferramentas disponíveis na plataforma *Google Drive* ^®^, onde foi armazenado o roteiro elaborado, em formato de texto não editável. Esta etapa ocorreu entre 04 de abril e 20 de maio de 2022 e permitiu a prévia avaliação dos especialistas, que analisaram o conteúdo do roteiro. Foram utilizados o IVCES^(13)^ e o instrumento para a caracterização dos especialistas*.*

Identificados os especialistas, foi encaminhada uma mensagem, por *e-mail*, via Plataforma Lattes^®^, convidando-os a participarem. A mensagem continha os endereços eletrônicos do Registro de Consentimento Livre e Esclarecido (RCLE), do roteiro do vídeo e do instrumento de coleta de dados. O acesso foi via *Google Forms* ^®^. Para os indicados pela técnica bola de neve, foi enviado *e-mail* com os mesmos acessos.

Os convites foram enviados quinzenalmente, exceto àqueles que informaram não ter interesse em contribuir com o estudo ou que já tinham respondido os instrumentos. No final, 23 especialistas responderam aos convites, compondo o comitê de especialistas*.* Não houve exclusões entre os que responderam.

Com base nos resultados atingidos na avaliação do roteiro, atualizou-se o roteiro do vídeo, em 15 dias. Para isso, foram consideradas as respostas dos especialistas na pergunta qualitativa aberta do instrumento de coleta de dados. As respostas foram confrontadas com a literatura e, quando convergentes, incluídas no roteiro.

A validação do vídeo - Fase 3 denominada pós-produção do vídeo também foi realizada em ambiente virtual e foram contatados os 23 especialistas que compuseram o comitê da primeira fase. Cada um recebeu cinco *e-mails* de convite, com *link* que direcionava ao *Google Forms* ^®^, contendo: um novo RCLE; o *link* do vídeo, disponível em formato não editável; e o Instrumento de Validação do Vídeo^(14)^. O comitê de avaliação do vídeo foi composto por 12 especialistas. A coleta de dados de validação do vídeo ocorreu entre 13 de abril e 06 de junho de 2023, totalizando 54 dias.

Após a implementação das sugestões realizadas, entre junho e agosto de 2023, pelos especialistas, o vídeo sobre a sondagem gástrica/enteral em Pediatria foi disponibilizado para o acesso público e gratuito no canal do *YouTube* ^®^ do Grupo de Pesquisa de Tecnologias, Gestão, Educação e Segurança no Trabalho (TeGEST), da UFCSPA.

### Tratamento e análise dos dados

Para a validação do roteiro e do vídeo foi utilizado o Google *Sheets* ^®^ Foi aplicado o Índice de Validade de Conteúdo (IVC), sendo considerados válidos os domínios e a avaliação global do IVCES ≥ 0,8. Foi utilizado o seguinte cálculo para o IVC: soma das respostas 2 / soma de todas as respostas^(15)^.

Para a caracterização dos especialistas, utilizou-se estatística descritiva por meio da média, frequência, desvio-padrão e moda. As respostas às questões abertas foram identificadas pela letra “E”, acrescido do número de ordenamento no banco de dados, por exemplo: “E1”, “E2”, […], “E23”.

Na fase 3, de pós-produção do vídeo, também foi adotado o ponto de corte de 0,80 e igual cálculo para o IVC, quando se utilizou o Instrumento de Validação do Vídeo. A mesma sistemática da fase 1 foi adotada, na fase 3, para a análise das questões abertas.

### Aspectos éticos

Este estudo teve a aprovação do Comitê de Ética em Pesquisa (CEP) da UFCSPA, nº 5.039.912 e utilizou-se o RCLE dos especialistas nas duas coletas de dados.

## Resultados

O estudo resultou em dois produtos: um roteiro e um vídeo sobre o procedimento de sondagem gástrica/enteral em Pediatria, voltado para os enfermeiros e os acadêmicos de Enfermagem.

Dos 23 enfermeiros que participaram do estudo, 21 (91,3%) eram do sexo feminino. A idade variou entre 34 e 68 anos, com média de 45,8±6,3 anos. No que se refere aos locais de residência dos especialistas, foram abrangidas três regiões brasileiras, sendo 10 (43,5%) especialistas da região Sul, 9 (39,1%), Sudeste e 4 (17,4%), Nordeste. Nas regiões Centro-Oeste e Norte não teve participação de especialistas. Obteve-se amostra de sete estados, com predomínio do estado do Rio Grande do Sul com 9 (39,1%) enfermeiros, São Paulo com 6 (26%) e o Rio de Janeiro com 3 (13%). Do Rio Grande do Norte foram 2 (8,6%) e Bahia, Pernambuco e Paraná com 1 (4,3%) para cada um dos estados.

O tempo de experiência profissional como enfermeiro variou entre oito e 45 anos, com média de 21,9±7,8 anos. A titulação máxima dos enfermeiros foi de 11 (47,8%) mestres, 10 (43,5%) doutores e 2 (8,7%) pós-doutores. Quanto à área de especialização houve predomínio da área de saúde da criança e do adolescente (14 - 60,9%), seguida da área de neonatologia (7 - 30,4%) e de paciente crítico (4 - 17,4%). Todos apresentaram experiência em Pediatria/saúde da criança, descritas no currículo.

Em relação ao tipo de instituição laboral, 12 (52,2%) atuavam em serviços na área da saúde e 11 (47,8%) na docência em ensino superior. Os 23 especialistas obtiveram uma pontuação, conforme os Critérios de Fehring, que variou entre oito e 12, com média de 11 pontos, e a moda e a mediana de 12.

O roteiro foi construído considerando 26 cenas. Elas abordaram conteúdos sobre a higiene das mãos, a identificação do paciente, a interação/vínculo com a família e a criança, os materiais necessários, o posicionamento da criança, a medição, a lubrificação, a inserção e a fixação da sonda, além dos testes para confirmar o posicionamento. A Tabela 1 mostra a avaliação dos especialistas em relação aos dezoito itens que compõem o IVCES.

**Tabela 1 -:** Validação do conteúdo do roteiro do vídeo, conforme o IVCES* (n = 23). Porto Alegre, RS, Brasil, 2022-2023

**Variáveis**	**IVC** ^†^
**Domínio 1 - Objetivos**	0,802
Contempla o tema proposto	0,820
Adequado ao processo de ensino-aprendizagem	0,900
Esclarece as dúvidas sobre o tema	0,562
Proporciona a reflexão sobre o tema	0,850
Incentiva a mudança de comportamento	0,833
**Domínio 2 - Estrutura/Apresentação**	0,816
Linguagem adequada ao público-alvo	0,930
Linguagem apropriada ao material educativo	0,904
Linguagem interativa, que permite o envolvimento ativo no processo educativo	0,833
Informações corretas	0,625
Informações objetivas	0,850
Informações esclarecedoras	0,625
Informações necessárias	0,722
Sequência lógica	0,820
Tema atual	0,878
Tamanho do texto adequado	0,878
**Domínio 3 - Relevância**	0,953
Estimula o aprendizado	0,954
Contribui para o conhecimento na área	0,954
Desperta o interesse pelo tema	0,952
**IVC** ^†^ **Global**	0,838

^*^IVCES = Instrumento de Validação de Conteúdo Educativo em Saúde; ^†^IVC = Índice de Validade de Conteúdo

No campo dissertativo, foi sugerido pelos especialistas (E2, E18 e E21) detalhar o cuidado centrado na criança e na família, incluindo os familiares como participantes ativos junto ao paciente, a fim de tornar o procedimento menos traumático. Também foi proposto incluir o brinquedo terapêutico como forma de facilitar na colaboração do paciente durante a inserção da sonda (E2, E18 e E21).

Três especialistas (E1, E13 e E17) propuseram abordar sobre a sondagem gástrica/enteral como atribuição privativa do enfermeiro, ao considerar a equipe de Enfermagem, e esclarecer as competências do enfermeiro na realização desse procedimento. Cinco especialistas (E1, E4, E14, E16 e E22) fizeram menção às especificidades do procedimento para os pacientes neonatais, apontando a necessidade de detalhar esses aspectos no material ou excluir esse público. Dois especialistas (E4 e E17) sugeriram substituir o termo “cateter” por “sonda”, devido à associação do primeiro termo à terapia intravenosa.

Dois especialistas (E19 e E20) propuseram maior atenção à marcação e à fixação da sonda, pois somente a marcação da caneta pode ser apagada, a fim de assegurar a fixação da sonda. Sugeriram que os autores devem seguir o padrão institucional, independentemente de a fixação estar localizada na bochecha ou nariz. Outra recomendação de inclusão foi o uso de placa de hidrocoloide para proteger a pele da criança para a fixação da sonda (E8, E17, E19 e E22).

Em relação aos testes de posicionamento do cateter, os enfermeiros (E2, E15 e E20) recomendaram a realização da radiografia, que é o padrão-ouro, pois a literatura aponta a contraindicação do teste de ausculta com o estetoscópio. Quanto ao volume de ar injetado para o teste de ausculta, três especialistas (E1, E18 e E19) ressaltaram a necessidade de descrever que há diferença do volume de ar injetado de acordo com a faixa etária. No que diz respeito à retirada do fio-guia ou mandril foi apontado que ela deve ser realizada após a inserção da sonda e antes do paciente ir para a radiografia (E2, E13, E18 e E20).

Como contribuições positivas, os especialistas avaliaram o material como “bastante didático” (E2) e que “está bem elaborado” (E22). Outros parabenizaram pela escolha do tema (E15) e consideraram “pertinente o tema, conteúdo e formato do roteiro” (E13). O roteiro também foi caracterizado como “primoroso e de excelente qualidade” (E21).

Após a avaliação e a validação realizada pelo comitê de especialistas, o roteiro foi atualizado. O vídeo foi gravado no Laboratório de Habilidades da UFCSPA, com sua versão inicial de duração 10 minutos e 1 segundo denominada “Sondagem gástrica/enteral em Pediatria”.

O vídeo tem cenas gravadas com manequim de simulação pediátrica, imagens de materiais usados no procedimento e telas com conteúdo teórico. Seu conteúdo abrange desde a preparação dos materiais até as técnicas específicas de inserção e verificação do posicionamento correto da sonda. Também apresenta diferentes recursos visuais, em formato de telas, para oferecer o suporte teórico e amparo legal. A Tabela 2 mostra a avaliação dos especialistas em relação aos 13 itens que compõem o instrumento para avaliação do vídeo.

**Tabela 2 -:** Validação do vídeo, conforme Instrumento de Validação de Vídeo (n = 12). Porto Alegre, RS, Brasil, 2022-2023

**Variáveis**	IVC*
**Domínio 1 – Funcionalidade**	0,909
O vídeo apresenta de forma clara e adequada os aspectos importantes do procedimento	0,909
O vídeo facilita o processo ensino-aprendizagem do procedimento	0,909
**Domínio 2 - Usabilidade**	0,896
No vídeo é fácil aprender os conceitos teóricos utilizados e a técnica do procedimento	0,857
O vídeo permite que os profissionais enfermeiros repliquem a técnica correta do procedimento na prática profissional	0,857
Sequência lógica	0,956
Tamanho do texto adequado	0,909
**Domínio 3 – Eficácia**	0,933
A duração do vídeo é adequada para aprender o conteúdo	0,909
O vídeo é atrativo/ prende a atenção do expectador	0,956
**Domínio 4 – Técnica Audiovisual**	0,985
A qualidade da imagem do vídeo é adequada para a observação das cenas	0,956
O tom e a voz dos narradores são claros e adequados	1,000
O vocabulário utilizado no vídeo é eficiente e compreensível ao público-alvo	1,000
**Domínio 5- Ambiente**	1,000
O ambiente utilizado para a gravação das cenas é considerado adequado	1,000
O material utilizado para a realização do procedimento está correto	1,000
IVC* Global	0,942

*IVC = Índice de Validade de Conteúdo

No campo descritivo, os especialistas (E1; E5; E7; E17 e E19) fizeram sugestões quanto à retirada do fio-guia e a forma de fixação e a mensuração da sonda no vídeo. Além disso, foi sugerido incluir que o médico é quem deve liberar o uso da sonda para a nutrição enteral (E17).

Quanto aos testes de posicionamento da sonda, foi sugerido detalhar melhor a técnica de ausculta da região epigástrica (E22); apontar que o teste de pH tem baixa acurácia por sofrer influência de substâncias do suco gástrico (E17); e que apesar de o raio-x ser padrão-ouro, deve ser utilizado com cautela (E5). Também, foi salientado que se deve utilizar mais de uma técnica para confirmar o correto posicionamento da sonda (E5 e E21).

Um especialista (E17) mencionou que o calibre da sonda utilizado para as crianças é a partir de 6 French. Para a escolha do calibre da sonda, devem ser consideradas as necessidades da criança a serem supridas pelo dispositivo (E5). Foi recomendado elencar os aspectos que diferenciam a sondagem gástrica e enteral, os tipos de sonda existentes, as contraindicações para a realização do procedimento e as orientações à família sobre o procedimento (E17 e E22).

Quanto ao recurso audiovisual, os especialistas E7 e E19 apontaram que o tempo de exibição de algumas telas informativas era curto. Sugeriram realizar a narração de todas as informações do material (E7); distribuir em mais telas as informações contidas em cada uma delas (E1) e mostrar a fixação da sonda e da retirada do mandril de um ângulo mais próximo (E19 e E21).

As sugestões realizadas pelos especialistas foram analisadas e confrontadas com a literatura para, então, serem incluídas. O vídeo foi atualizado e a versão final contém duração de 10 minutos e 10 segundos. Após, o vídeo foi disponibilizado no canal do *YouTube* ^®^ do Grupo de Pesquisa, pelo *link* https://bit.ly/47Suc7l.

## Discussão

O predomínio de enfermeiras na idade adulta média entre os especialistas reflete os dados de uma pesquisa envolvendo a profissão no Brasil, em que 86,2% são mulheres e que 66,6% têm idade superior a 40 anos^(16)^. A amostra contemplou enfermeiros especialistas de três das cinco regiões do Brasil, não havendo avaliadores do Centro-Oeste e do Norte. Isso corrobora os dados da pesquisa brasileira que mostra uma menor concentração de enfermeiros nessas duas regiões do país, sendo de 8,2% e 6,7%, respectivamente^(16)^.

Ao analisar a Unidade Federativa dos participantes, o estado do Rio Grande do Sul representou 39,1% da amostra. É provável que o número mais expressivo de enfermeiros deste estado se justifique pela origem do estudo.

O tempo de experiência profissional e a titulação dos especialistas se assemelham aos de outros estudos que validaram materiais educativos, em que se exige maior titulação e especialização^(17 - 22)^. A pontuação média de Fehring (adaptada) foi 11, variando de oito a 12 pontos. Portanto, esses resultados evidenciaram o perfil distinto dos participantes deste estudo, validando, portanto, a especialização do comitê.

Na construção do roteiro/vídeo, buscou-se apresentar conteúdos teóricos relacionados ao procedimento de sondagem gástrica/enteral, como: conceito, as indicações/contraindicações, o objetivo, o amparo legal, os materiais necessários e as técnicas lúdicas. Além disso, abordaram-se, também, alguns pontos controversos na realização do procedimento, como medida do comprimento e do diâmetro da sonda, a posição/contenção da criança, a estabilização do dispositivo e os testes de confirmação de posicionamento.

Nos últimos cinco anos houve um crescimento na produção de vídeos educativos no ensino de Enfermagem, potencializado pela explosão digital, pelas recentes demandas de ensino e mudanças no perfil dos alunos, com a inserção de novas ferramentas educacionais. Uma revisão integrativa, publicada em 2023, encontrou 19 artigos sobre a produção de vídeos na Enfermagem, e a maioria utilizou as fases de pré-produção, produção, pós-produção, com a validação por especialistas, como ocorreu nesta investigação^(9)^.

Esse método de construção tem apresentado resultados promissores quando analisado o desfecho do vídeo na população-alvo. Um Ensaio Clínico Randomizado (ECR) que buscou avaliar a efetividade de um vídeo educativo no conhecimento de 100 pacientes hospitalizados sobre as práticas seguras no período perioperatório identificou um ganho de conhecimento significativamente superior (*t* =3.72 ±1,84; *p*<0,001) no grupo intervenção (vídeo educativo) ao grupo controle (orientações-padrão)^(23)^.

Outro estudo mostrou que os acadêmicos de Enfermagem lusófonos tiveram maior aquisição de conhecimento sobre a punção venosa periférica após a utilização de um vídeo educativo validado sobre a temática^(24)^. Logo, a proposta de construção e de validação de vídeos educativos voltados às situações da assistência de Enfermagem pode favorecer o desenvolvimento de conhecimentos e habilidades dos profissionais^(25 - 26)^, como propõe este estudo. Quando não avaliado por especialistas, o material oferecido pode conter erros de conteúdo e ter um *design* pouco atrativo, o que pode desmotivar o uso pelo público-alvo e estimular as práticas inadequadas^(27)^.

O roteiro do vídeo sobre a sondagem gástrica/enteral em crianças atingiu o IVC global 0,838 e o vídeo IVC global 0,942, com cada domínio variando de 0,8002 a 0,952 (roteiro) e 0,896 a 1,0 (vídeo). Desse modo, ambos os produtos são considerados adequados^(15)^. Outros estudos que validaram os vídeos educativos, com diferentes temas, populações e instrumentos, também utilizaram o ponto de corte 0,8 para o IVC^(23 , 25 , 28 - 30)^.

Um estudo metodológico que construiu e validou um vídeo educativo para os estudantes de Enfermagem sobre a parada cardiorrespiratória obstétrica obteve IVC global de 0,99, com os domínios do IVCES variando de 0,95 a 1^(28)^. Outra investigação, com o mesmo instrumento, validou sete vídeos educativos para a promoção da saúde e a segurança no trabalho de profissionais que atuam na Atenção Primária à Saúde, com o domínio global entre 0,88 e 0,96. No domínio “objetivos” o IVC variou de 0,84 a 0,95; em “estrutura/apresentação” de 0,91 a 0,99; e “relevância” de 0,81 a 0,95^(25)^ semelhantes a este estudo.

A validação do roteiro acrescentou qualidade ao conteúdo para a posterior elaboração do vídeo, que apresentou IVC superior ao roteiro. As contribuições dos especialistas que avaliaram o roteiro e o vídeo foram essenciais para aprimorar as informações e garantir a qualidade dos produtos construídos, sobretudo em conteúdos controversos, como medida do comprimento da sonda e testes confirmatórios para o posicionamento. Como as sugestões foram pontuais, o que foi permitido pelas questões abertas dos instrumentos, e todos os valores de IVC dos domínios dos dois instrumentos foram superiores ao ponto de corte estabelecido (0,80), manteve-se uma rodada de avaliação e as alterações foram realizadas com a análise dos comentários, confronto com a literatura^(31 - 34)^ e a decisão final dos autores.

Na validação do roteiro do vídeo, foram realizadas observações sobre os objetivos do procedimento e sobre a necessidade de inclusão de resoluções do Conselho Federal de Enfermagem (COFEN) no procedimento de sondagem gástrica/enteral e na contenção mecânica do paciente, as quais foram acrescentadas. Os especialistas também apontaram a necessidade de enfatizar o cuidado centrado na criança e na família, a fim de tornar menos traumáticos a internação hospitalar e os procedimentos realizados nesse período. Para prestar um cuidado integral ao paciente pediátrico, deve-se explicar claramente o procedimento à criança, de acordo com o seu estágio de desenvolvimento, e aos seus familiares, incluindo-os no cuidado. Uma forma de executar isso é com o uso do brinquedo terapêutico, um instrumento atrativo para a criança que permita demonstrar de forma lúdica o que será realizado, possibilitando um procedimento mais tranquilo e humanizado^(35)^.

Em um primeiro momento, no roteiro construído, não havia distinção do procedimento em atendimento as diferentes faixas etárias da Pediatria. Devido a isso, foi salientada pelos especialistas a importância de realizar a abordagem das especificidades do período neonatal. Esse período é caracterizado do nascimento até o 28º dia de vida, sendo uma fase restrita^(36)^. Desse modo, ao verificar as particularidades dessa fase do desenvolvimento, optou-se por não contemplar esse público no material, visto que a maioria do público-alvo se concentra após o 28º dia de vida. Essa decisão foi informada no roteiro final e no vídeo.

Alguns especialistas citaram a necessidade de trocar o termo cateter, usado inicialmente para a elaboração do roteiro, pelo termo sonda, tradicional e historicamente utilizado nos serviços de saúde e no ambiente universitário. Optou-se por trocar o termo “cateter” por “sonda”, por este ser o mais utilizado no país, embora tecnicamente o uso de “cateter” ou “tubo” sejam adequados, e esse último o mais utilizado em publicações na língua inglesa^(1 - 2 , 5 - 7 , 31 - 33)^.

No procedimento de sondagem gástrica/enteral, após a sua realização, é necessária a utilização de cuidados, tais como o de garantir a localização adequada do dispositivo no trato gastrointestinal. Quanto a isso, os especialistas referiram que as melhores práticas não mais indicavam a realização do teste de ausculta da região epigástrica, e sim a necessidade da radiografia. Alguns autores não recomendam e desencorajam o uso do teste da ausculta devido à possibilidade de auscultar os ruídos na região epigástrica, independente se a ponta da sonda está localizada no estômago, esôfago ou no trato respiratório^(31)^. Em consonância com as contribuições dos especialistas e os achados na literatura^(31 - 33)^, foram ressaltados esses dois aspectos no roteiro quando se trata dos testes para confirmar o posicionamento da sonda.

Na literatura, há divergências quanto à acurácia dos métodos. Devido a isso, foi salientada no vídeo a relevância de sempre realizar duas técnicas à beira-leito para confirmar o posicionamento correto da sonda antes de utilizá-la^(31 - 33)^. O raio-x é considerado o padrão-ouro para a confirmação do posicionamento da sonda, entretanto, como mencionado pelo avaliador e incorporado ao vídeo, deve ser utilizado com cautela, já que a exposição repetitiva à radiação pode ser perigosa para a criança^(31 - 33)^.

A acurácia do teste de pH foi questionada por uma especialista, pois o pH pode ser modificado pela administração da dieta recente e o uso de medicamento inibidor da secreção gástrica. A literatura mostra que não há diferença significativa entre os pacientes que receberam o medicamento ou o alimento e os que não receberam, mas pode causar confusão ao profissional, sendo importante utilizar uma segunda técnica para a confirmação do posicionamento^(31 - 33)^. Diante disso, mantiveram-se as orientações sobre o teste de pH no vídeo, pois este pode ser considerado um teste sensível para a identificação do posicionamento da sonda^(31 - 33)^.

Um especialista apontou a necessidade de descrever melhor o teste de ausculta da região epigástrica. Essa técnica estava descrita no roteiro do vídeo, mas não havia sido incorporada ao vídeo em formato de texto, por ter sido demonstrada em filmagem. A fim de esclarecer a informação, foi acrescentada uma descrição do procedimento no vídeo.

Outro cuidado importante para garantir o conforto do paciente refere-se à pele e às mucosas. Em vista disso, alguns especialistas salientaram a importância da utilização de placas de hidrocoloide na pre-fixação da sonda. Essa característica foi acatada nas correções realizadas no roteiro, ao considerar que esta tecnologia reduz a umidade, o atrito e o cisalhamento, indicado para evitar as lesões por pressão, inclusive aquelas relacionadas ao uso de dispositivos médicos^(37)^.

Entretanto, vale salientar que, por se tratar de um material de custo elevado, ele nem sempre será de fácil acesso nos níveis de atenção e/ou serviços, ainda mais considerando as diferenças dos serviços de saúde no Brasil.

Na validação do roteiro e, posteriormente, do vídeo, os especialistas abordaram as diferentes maneiras de se realizar a fixação/estabilização da sonda, ou seja, utilizar barbante ou estabilizar no buço, no queixo ou na região temporomandibular. Devido às diferentes orientações, manteve-se o que já estava no roteiro inicial, uma vez que não existe um padrão-ouro para a realização e deve-se considerar as peculiaridades do paciente e seguir as orientações da instituição de atuação profissional, sempre prezando pela segurança e o conforto do paciente ao garantir os cuidados com a pele. Foi incluída, no roteiro, uma observação quanto à fixação da sonda quando inserida oralmente, o que não havia sido especificado anteriormente. Nesse estudo, considerou-se que a sonda deve ser fixada, utilizando a fita adesiva microporosa, à bochecha da criança e ao nariz, se colocado através das narinas^(38)^. Quando inserida por via oral, a sonda deve ser posicionada centralmente e estabilizada acima do lábio superior, quando possível^(39)^.

Ainda há divergência de saberes e lacunas na literatura quanto à forma de realizar a inserção e a manutenção da sonda gástrica/enteral. Entende-se que é preciso aplicar o rigor técnico-científico e que a segurança do paciente seja garantida em todas as etapas do procedimento. Um exemplo dessa divergência é o momento da retirada do fio-guia, apontada pelos especialistas que avaliaram o roteiro e o vídeo, os quais realizaram as diferentes recomendações a esse respeito. Alguns autores recomendam a retirada após a realização do raio-x^(38)^, outros, por sua vez, orientam que pode ser retirado imediatamente após a inserção da sonda e a estabilização do dispositivo na pele, o que pode favorecer a migração da ponta da sonda e o seu posicionamento final antes mesmo da realização do exame de imagem^(40)^. Neste estudo, optou-se por adotar a recomendação de retirar o fio-guia após a realização dos testes de posicionamento e antes de encaminhar o paciente para o raio-x ^(34 , 40)^.

Na validação do vídeo, foi recomendado revisar a forma de mensuração do comprimento a ser inserido da sonda. Diversas recomendações são descritas na literatura, como a mensuração baseada nos aspectos morfológicos NEX (*Nose*, *Earlobe*, *Xiphoid*) e NEMU (*Nose*, *Earlobe*, *Mid-Umbilicus*) para a sondagem gástrica e o método NEX, acrescido de quatro centímetros para a sondagem enteral^(31 , 33)^. Além disso, os especialistas ressaltaram a possibilidade de mensuração com base na relação idade e altura. Optou-se em manter a forma utilizada no vídeo, sendo a medida da posição gástrica da ponta do nariz/borda da boca ao lóbulo da orelha, e deste ao espaço médio entre o apêndice xifoide e a cicatriz umbilical; e a da posição entérica da ponta do nariz ao lóbulo da orelha, deste ao apêndice xifoide e depois até a cicatriz umbilical^(34)^.

Os especialistas também realizaram as sugestões sobre o calibre da sonda, que foram implementadas no vídeo. A primeira refere-se ao fato de que se pode utilizar sondas a partir de 6 French em crianças, como apontado pela literatura^(34)^, sendo que no vídeo orientava-se a partir de 6 French^(36)^. Salienta-se que, ao escolher o calibre da sonda, deve-se optar pela de menor diâmetro, considerando as características da criança e as necessidades terapêuticas a serem supridas pelo dispositivo.

Algumas informações sugeridas não foram acrescentadas, como: contraindicações para a realização do procedimento e os aspectos que diferenciam a sondagem gástrica e enteral, a fim de se evitar um vídeo longo. No entanto, elas estão disponibilizadas como material complementar do vídeo, acessado por *Quick Responde Code* e/ou na descrição do vídeo no *YouTube* ^®^. Sobre o tempo de duração (10 minutos e 10 segundos), acredita-se que ele contribua para o envolvimento e o aprendizado do espectador, respeitando as recomendações que estipulam que sejam de seis a 15 minutos^(41 - 42)^, similarmente a outros estudos que validaram os vídeos educativos para a Enfermagem^(28 - 30 , 43 - 44)^.

Quanto à técnica audiovisual, os autores do vídeo optaram por aumentar o tempo de exibição de algumas telas informativas, a fim de permitir a leitura sem ser necessário pausar o vídeo. Além disso, foi alterado o ângulo de gravação da fixação da sonda e da retirada do mandril, como recomendado. No entanto, não foi acatada a sugestão sobre a narração de todas as informações do material.

O roteiro e o vídeo, elaborados com rigor científico e validados por especialistas, podem ser utilizados como uma estratégia para diversificar os processos de ensino-aprendizagem dos enfermeiros e acadêmicos de Enfermagem em sala de aula, em laboratório ou em atividades complementares. O vídeo, intitulado “Sondagem gástrica/enteral em Pediatria”, construído com base no roteiro, pode ser utilizado pelo profissional/aluno durante a realização do procedimento, sendo de fácil consulta, especialmente por estar disponível em plataformas de acesso livre na *Internet*, como o *YouTube* ^®^. O roteiro pode ser acessado por meio do *link* disponibilizado na descrição do vídeo, bem como os outros materiais que corroboraram sua elaboração.

Os resultados e o produto (vídeo) são originais e inéditos, pois se desconhece a publicação de estudo sobre a elaboração de um vídeo educativo sobre o público-alvo e a temática. Assim, ressalta-se sua originalidade e potencialidade para auxiliar os profissionais no desenvolvimento teórico-prático das competências na temática do procedimento sobre a sondagem gástrica e enteral em crianças.

Como limitação, tem-se a maior participação loco-regional dos especialistas que realizaram a validação dos produtos, o que pode limitar o uso dos produtos nas Regiões Centro-Oeste e Norte do Brasil, em virtude das diferenças culturais no país. A perda de 11 especialistas na última etapa não invalida os resultados, visto que os 12 avaliadores do vídeo ultrapassaram o mínimo estipulado pela literatura, que são seis^(15)^. Como não foram encontradas outras investigações envolvendo o tema e o público-alvo, o que exalta a sua originalidade, há restrição no confronto dos dados com outros estudos. Por fim, o uso do IVC, fundamental no processo de desenvolvimento de produtos, apresenta como limitação a subjetividade dos especialistas ^(15)^, o que se buscou minimizar com as decisões embasadas na literatura.

## Conclusão

O vídeo “Sondagem gástrica/enteral em Pediatria” foi validado pelo comitê de especialistas. O estudo contribui com o avanço do conhecimento científico e para a prática assistencial do enfermeiro ao proporcionar um recurso educativo que contém conteúdo elaborado com rigor científico, de fácil acesso e gratuito.

O vídeo pode ser utilizado de maneira independente em diferentes propostas pedagógicas, desde que seja cumprida a licença *Creative Commons* Atribuição 4.0 Internacional. Sugere-se a condução de estudos posteriores para a análise do seu efeito no conhecimento e nas habilidades de enfermeiros e acadêmicos.

# Translation: Creación y validación de un video sobre la inserción de sondas gástricas y enterales en niños*

**DOI:** 10.1590/1518-8345.7226.4505

**Authors:** Gabriela Beatriz Leonhardt, Giovani Basso da Silva, Guilherme Kayser Prates, Simone Travi Canabarro, Luccas Melo de Souza

**Affiliations:** 1 Santa Casa de Misericórdia de Porto Alegre, Hospital da Criança Santo Antônio, Porto Alegre, RS, Brasil.; 2 Becaria (o) de la Fundação de Amparo à Pesquisa do Rio Grande do Sul (FAPERGS), Brasil.; 3 Santa Casa de Misericórdia de Porto Alegre, Hospital Santa Rita, Porto Alegre, RS, Brasil.; 4 Universidade Federal de Ciências da Saúde de Porto Alegre, Porto Alegre, RS, Brasil.; 5 Universidade Federal de Ciências da Saúde de Porto Alegre, Departamento de Enfermagem, Porto Alegre, RS, Brasil.

**Keywords:** Nutrición Enteral, Salud Infantil, Enfermería, Educación en Enfermería, Investigación Metodológica en Enfermería, Película y Video Educativos

## Abstract

crear y validar un video educativo sobre el procedimiento de inserción de sondas gástricas y enterales en niños.

estudio metodológico realizado en tres fases: a) preproducción del video; b) producción; y c) postproducción. Primero, se creó un guion basado en la literatura científica. El video fue grabado en el Laboratorio de Simulación y editado utilizando el *software Movavi* ^®^. El guion y el video fueron evaluados por 23 y 12 expertos, respectivamente, a través de *Google Forms* ^®^. Se utilizaron los instrumentos Validación de Contenido Educativo en Salud, Validación de Video e Índice de Validez de Contenido, con punto de corte ≥ 0,80.

los expertos representaron tres regiones del país. El video contiene escenas grabadas con un maniquí de simulación pediátrica, imágenes de los materiales utilizados en el procedimiento y pantallas con contenido teórico. El guion del video obtuvo un índice de validación de contenido total de 0,83 y el video de 0,94, ambos en una sola ronda. El video final tiene una duración de 10 minutos y 10 segundos.

el guion y el video fueron validados. Está disponible de forma gratuita en *YouTube* ^®^ y puede ser utilizado por enfermeros/estudiantes de enfermería como instrumento de estudio y atención profesional.

## Destaques

**(1)** Hay que utilizar metodología científica para crear videos educativos de enfermería. **(2)** El guion del video y el video fueron validados por expertos. **(3)** El material fue catalogado como extraordinario y de excelente calidad. **(4)** El video es de libre acceso y puede contribuir al desarrollo de prácticas cualificadas y seguras.

## Introducción

El cateterismo gástrico y enteral son procedimientos de rutina que realizan los enfermeros, especialmente en el contexto hospitalario, en pacientes de todas las edades^(1 - 2)^. Consiste en introducir una sonda (o tubo) a través de la fosa nasal o la boca hasta el estómago (sonda nasogástrica u orogástrica) o el intestino (sonda nasoentérica u oroentérica). Hay pocos datos epidemiológicos sobre el uso de estas sondas, especialmente en niños. En 2016, un estudio multicéntrico identificó que se utilizó sonda gástrica o enteral en el 24 % de los 8333 pacientes pediátricos/neonatales ingresados en 63 hospitales de Estados Unidos da América. Había 1.316 sondas nasogástricas (66%), 414 sondas orogástricas (21%) y 261 sondas enterales (17%)^(3)^.

En la atención infantil, es común utilizar sondas gástricas y enterales como soporte nutricional cuando no se puede realizar por vía oral. La finalidad/indicación de las mismas es administrar dieta y/o medicación, hidratación, además permiten realizar lavado, drenaje de líquidos o aire y recolección de material gástrico^(4 - 5)^. Las contraindicaciones incluyen obstrucciones intestinales, perforaciones gastrointestinales y determinadas anomalías congénitas que imposibilitan la intervención^(1 , 5)^. La elección de la colocación de la sonda, ya sea gástrica o enteral, depende de la condición clínica del paciente y de la necesidad específica de la terapia, se consideran la duración del uso, el riesgo de aspiración y la necesidad de alimentación continua^(1 , 6)^.

La inserción o el uso inadecuado de estos dispositivos puede provocar eventos adversos, como molestias nasofaríngeas, vómitos, epistaxis, erosión del tabique nasal, lesiones por presión relacionadas con la fijación, complicaciones respiratorias e incluso la muerte^(1 , 5)^. Un estudio realizado con 130 enfermeros en Arabia Saudita demostró que más de la mitad no tenía conocimiento suficiente (53,1%) o eran incompetentes (58,5%) al momento de insertar sondas gástricas o enterales^(7)^.

Por eso, es fundamental que las acciones del equipo de Enfermería tengan como objetivo calificar los procesos de atención y garantizar la seguridad infantil, y que se basen en las mejores prácticas de atención. En este caso, la Educación Permanente en Salud (EPS) para profesionales surge como una estrategia esencial para mejorar la atención a los niños que necesitan estas sondas, ya que promueve la actualización constante del conocimiento y evita que las prácticas se vuelvan obsoletas en un escenario de rápida evolución tecnológica y científica^(8 - 9)^.

Una de las tecnologías que ha sido ampliamente utilizada en la enseñanza de Enfermería en los últimos años es el video educativo, y es una estrategia que sirve para difundir rápidamente información sobre contenidos/procedimientos. Permite sintetizar y visualizar la teoría y asociarla con la práctica, le agrega dinamismo al contenido, potencia la construcción del conocimiento, la comprensión y la reflexión, siempre y cuando se desarrolle adecuadamente, es decir, con rigor metodológico^(8 - 9)^.

Dada la complejidad de la inserción de sondas gástricas y enterales, la constante evolución del conocimiento científico y de las prácticas asistenciales, así como las divergencias en la ejecución del procedimiento^(7)^, se consideró importante crear un video educativo que oriente la estandarización de los pasos de este procedimiento, con el objetivo de llenar vacíos en la educación continua de los profesionales de la salud. Este tipo de recurso educativo puede contribuir al proceso de enseñanza-aprendizaje y calificar la atención en salud, dependiendo del método y marco teórico utilizado^(8)^. Por lo tanto, este estudio tuvo como objetivo crear y validar un video educativo sobre el procedimiento de inserción de sondas gástrica y enteral en niños.

## Método

### Diseño del estudio

Se trata de un estudio metodológico, con producción de tecnología educativa en formato de video. Para su estructuración se siguieron las recomendaciones de la guía *Revised Standards for Quality Improvement Reporting Excellence* (SQUIRE 2).

Se utilizó la guía de creación de videos de Fleming, Reynolds y Wallace^(10)^, uno de los métodos más utilizados para este tipo de producción en Enfermería^(9)^. Se realizó en tres fases: a) preproducción del video; b) producción y c) postproducción. En la fase 1, el guion del video fue creado y validado por expertos. La fase 2 constituyó la grabación de video. En la fase 3, el video pasó por la validación de expertos, ajustes finales y fue publicado.

La fase 1, preproducción del video, se realizó en cuatro etapas (búsqueda bibliográfica, elaboración, validación y actualización del guion), con el fin de elaborar el guion de producción. Al no existir consenso ni *guidelines* sobre la técnica de inserción y con el objetivo de identificar las mejores prácticas en la inserción de sonda gástrica/enteral en Pediatría, la búsqueda del contenido teórico para la elaboración del guion se realizó mediante una revisión narrativa de la literatura en las siguientes bases de datos: *Cumulative Index to Nursing and Allied Health Literature* (CINAHL), *Medical Literature Analysis and Retrieval System Online* (MEDLINE), *SciVerse Scopus* (SCOPUS) y *Web of Science*. Se utilizó literatura gris, se llevó a cabo una búsqueda en el catálogo de Tesis y Disertaciones de la Coordinación de Perfeccionamiento del Personal de Educación Superior (CAPES) y en libros de referencia en el área de Pediatría.

Después de seleccionar la información relevante para componer el material, se organizó el contenido del guion y se puesto en un cuadro. Cada línea correspondía a una escena del video y las tres columnas contenían la siguiente información: escena, descripción del contenido de la escena y descripción técnica de la escena^(11)^. El guion fue elaborado por dos enfermeros con doctorado y una estudiante de enfermería, todos autores del estudio.

En la columna “descripción del contenido de la escena” se especificaron las acciones que realizaba la enfermera, la técnica para la realización del procedimiento, los textos o discursos. En la “descripción técnica de la escena” se abordaron aspectos visuales, como imágenes, audio y/o video, con base en estudios preexistentes^(11)^. El guion se elaboró en 42 días.

En la fase 2, producción del, previa modificación del guion, se gravaron las escenas y las narraciones descritas en la preproducción. Se seleccionó material, como artículos, documentos, entre otros, para componer el producto y crear pantallas con información adicional, realizadas con el *software Canva* ^®^. Para editar el video se utilizó el *softwareMovavi* ^®^.

Se realizaron ajustes en las grabaciones de las escenas, también se incluyó en un solo producto las escenas grabadas, la narración y las pantallas informativas. Esta fase se desarrolló entre el 30 de junio de 2022 y el 17 de marzo de 2023 (260 días) y participaron tres investigadores en la grabación y dos en la edición. Todo el equipo (dos profesores asesores y tres estudiantes de grado) participó en el análisis inicial del video.

### Lugar del estudio

La elaboración del guion (Fase 1) y del video (Fase 2) tuvo lugar en los laboratorios de enseñanza de la Universidad Federal de Ciencias de la Salud de Porto Alegre (UFCSPA), en Porto Alegre, Rio Grande do Sul, Brasil, con profesores y becarios de iniciación científica de la carrera de Licenciatura en Enfermería. Esta universidad cuenta con 16 carreras de grado, todas vinculadas al área de la salud. La validación de los materiales se realizó en un entorno virtual, utilizando la Plataforma Lattes^®^ y las herramientas de *Google Drive* ^®^ y *Google Forms* ^®^.

### Periodo

El estudio se realizó entre marzo de 2022 y agosto de 2023.

### Población

Para la fase de validación del guion y del video, se reclutaron expertos en todo Brasil, a través de la Plataforma Lattes^®^, que reúne, de manera abierta, CV de investigadores (Currículo Lattes) e información de grupos de investigación e instituciones del país.

### Criterios de selección

En la fase 1, para validar el guion del video, se reclutó a los expertos de dos formas. Los primeros mediante una búsqueda en la Plataforma Lattes^®^, utilizando las siguientes palabras clave y operadores booleanos: *enfermagem and intubação gastrointestinal and pediatria or saúde da criança; enfermagem and nutrição enteral and pediatria or saúde da criança*. A los expertos que respondieron el instrumento de recolección de datos se les solicitó que indicaran hasta otros tres investigadores para participar en la validación del material pela técnica de bola de nieve. Se enviaron 101 invitaciones, es decir 66 a través de la Plataforma Lattes^®^ y 35 mediante la técnica de bola de nieve.

Los criterios de inclusión de los expertos fueron: ser enfermero, trabajar en Brasil y alcanzar al menos cinco puntos en los Criterios de Fehring^(12)^ adaptados, considerando: maestría y/o doctorado en Enfermería (cuatro puntos); artículo publicado en el área de interés (tres puntos); capacitación (especialización o curso) en el área de interés (dos puntos); práctica profesional (asistencial docencia o investigación) de dos años o más en el área de interés; disertación/tesis en el área de interés (un punto). El área de interés se definió como “salud infantil/pediatría”. Como criterio de exclusión se adoptó el siguiente: no tener como lengua materna el portugués brasileño, ya que ese es el idioma del video.

### Definición de la muestra

La muestra se estipuló considerando un mínimo de seis y un máximo de 20 expertos, que es la que se considera suficiente^(9)^. Luego de enviar los correos electrónicos y llegar a los 20 expertos, otros tres completaron la evaluación, por ende, la muestra para evaluar el guion estuvo compuesta por 23 enfermeros expertos.

### Variables de estudio

En lo que respecta a los expertos, se recolectaron las siguientes variables: edad, sexo, ciudad/estado de actividad, tiempo de experiencia profesional en Enfermería, formación/título profesional, área de especialización y lugar de ejercicio profesional actual. En cuanto a la validación del guion y del video, las variables incluyeron aspectos relacionados con los objetos de estudio, es decir, validación del guion y del video, recolectados mediante instrumentos específicos.

### Instrumentos utilizados para la recolección de datos

Los instrumentos de recolección de datos se insertaron en *Google Forms* ^®^. Para evaluar el guion se utilizó el Instrumento de Validación de Contenidos Educativos en Salud (IVCES)^(13)^, que contiene 18 preguntas organizadas en tres dominios: objetivos, estructura/presentación y relevancia. Las preguntas del IVCES tienen un formato de escala Likert, con tres opciones de respuesta (0 = en desacuerdo, 1 = parcialmente de acuerdo y 2 = completamente de acuerdo). Se incluyeron ocho preguntas para caracterizar a los expertos y una pregunta descriptiva para críticas y recomendaciones de forma libre.

Para la validación del video se adaptó el Instrumento de Validación del Video utilizado en un estudio anterior^(14)^. Contiene cinco dimensiones que analizan la funcionalidad, la usabilidad, la eficiencia, la técnica audiovisual y el entorno. Este instrumento también presenta una escala Likert, con tres opciones de respuesta (0 = en desacuerdo, 1 = parcialmente de acuerdo y 2 = completamente de acuerdo) para los 11 ítems evaluados.

### Recolección de datos

La validación del guion de la video - fase 1, preproducción, se realizó en un entorno virtual, utilizando las herramientas disponibles en la plataforma *Google Drive* ^®^, donde se almacenó el guion elaborado en formato de texto no editable. Esta etapa, realizada entre el 04 de abril y el 20 puede de 2022, permitió la realización de una evaluación previa por parte de expertos, quienes analizaron el contenido del guion. Para caracterizar a los expertos se utilizó el IVCES^(13)^ y el instrumento.

Una vez identificados los expertos, se les envió un mensaje, por correo electrónico, a través de la Plataforma Lattes^®^, invitándolos a participar. El mensaje contenía las direcciones electrónicas del Registro de Consentimiento Libre e Informado (RCLI), del guion del video y del instrumento de recolección de datos. El acceso se llevó a cabo mediante *Google Forms* ^®^. A los indicados mediante la técnica de la bola de nieve se les envió un correo electrónico con los mismos accesos.

Las invitaciones se enviaron cada quince días, excepto a aquellos que indicaron que no tenían interés en contribuir al estudio o que ya habían respondido. Al final, 23 expertos respondieron las invitaciones y conformaron el comité de expertos. No hubo exclusiones entre quienes respondieron.

En base a los resultados alcanzados en la evaluación del guion, se actualizó el guion del video (en 15 días). Para ello, se consideraron las respuestas de los expertos a la pregunta cualitativa (abierta) del instrumento de recolección de datos. Las respuestas se compararon con la literatura y, cuando convergieron, se las incluyó en el guion.

También se realizó la validación del video - fase 3, postproducción del video, en un entorno virtual y se contactó a los 23 expertos que integraron el comité de la primera fase. Cada persona recibió cinco correos electrónicos de invitación, con un *link* a *Google Forms* ^®^, que contenían: una nueva RCLI; el *link* del video, disponible en formato no editable; y el Instrumento de Validación del Video^(14)^. El comité de evaluación del video estuvo conformado por 12 expertos. La recolección de datos de validación del video se llevó a cabo entre el 13 de abril 2023 y el 6 de junio de 2023 (54 días).

Luego de implementar las sugerencias de los expertos (realizadas entre junio y agosto de 2023), el video sobre cateterismo gástrico/enteral en Pediatría se subió al canal de *YouTube* ^®^ del Programa de Tecnología, Gestión, Educación y Seguridad en el Trabajo (TeGEST), de la UFCSPA para que el público accediera de forma gratuita.

### Procesamiento y análisis de datos

El análisis se realizó en *Google Sheets* ^®^ para validar el guion y el video. Se aplicó el Índice de Validez de Contenido (IVC) y se consideraron validados los dominios y la evaluación total del IVCES cuando ≥ 0,8. Para el IVC se utilizó el siguiente cálculo: suma de las respuestas 2 / suma de todas las respuestas^(15)^.

Para caracterizar a los expertos se utilizó estadística descriptiva (media, frecuencia, desviación estándar y moda). Para analizar y presentar las respuestas de las preguntas abiertas, las mismas se identificaron con la letra “E”, más el número de orden en la base de datos, por ejemplo: “E1”, “E2”, […], “E23”.

En la fase 3, postproducción del video, también se adoptó un punto de corte de 0,80 y el mismo cálculo para el IVC, al utilizar el Instrumento de Validación del Video. Se adoptó en la fase 3 la misma metodología que en la fase 1, para analizar las preguntas abiertas.

### Aspectos éticos

Este estudio fue aprobado por el Comité de Ética en Investigación (CEP) de la UFCSPA, nº 5.039.912 y en ambas recolecciones de datos se utilizó el RCLI con los expertos.

## Resultados

El estudio dio como resultado dos productos: un guion y un video sobre el procedimiento de cateterismo gástrico/enteral en Pediatría, dirigidos a enfermeros y estudiantes de enfermería.

De los 23 enfermeros que participaron en el estudio, 21 (91,3%) eran del sexo femenino. La edad varió entre 34 y 68 años, con una media de 45,8±6,3 años. En cuanto a los lugares de residencia de los expertos, representaron tres regiones brasileñas, 10 (43,5%) expertos eran del Sur, 9 (39,1%) del Sudeste y 4 (17,4%) del Nordeste. No había expertos de las regiones Centro-Oeste y Norte. Se obtuvo una muestra de siete estados, predominó Rio Grande do Sul con 9 (39,1%) personas, São Paulo con 6 (26%) y Río de Janeiro con 3 (13%). Dos 2 (8,6%) expertos eran de Rio Grande do Norte y 1 (4,3%) de cada uno de los estados de Bahía, Pernambuco y Paraná.

El tiempo de experiencia profesional como enfermero osciló entre ocho y 45 años, con una media de 21,9±7,8 años. El mayor título que poseían los expertos era 11 (47,8%) máster, 10 (43,5%) doctorado y dos 2 (8,7%) postdoctorado. En cuanto al área de especialización, predominó la salud infanto-juvenil 14 (60,9%), seguida del área de neonatología 7 (30,4%) y pacientes críticos 4 (17,4%). Todos con experiencia en Pediatría/salud infantil, según el análisis del CV.

En lo que respecta al tipo de institución de trabajo, 12 (52,2%) trabajaban en servicios de salud y 11 (47,8%) en la docencia en Educación Superior. Los 23 expertos obtuvieron una puntuación, según los Criterios de Fehring, que osciló entre ocho y 12, con una media de 11 puntos, y una moda y mediana de 12.

El guion se elaboró incluyendo 26 escenas. Abarcaron contenidos sobre higiene de manos, identificación del paciente, interacción/vínculo con la familia y el niño, materiales necesarios, posicionamiento del niño, medición, lubricación, inserción y fijación de la sonda, además de pruebas para confirmar el posicionamiento. La Tabla 1 muestra la evaluación de los expertos de los dieciocho ítems que componen el IVCES.

**Tabla 1 -:** Validación del contenido del guion del video, según el IVCES* (n = 23). Porto Alegre, RS, Brasil, 2022-2023

**Variables**	**IVC** ^†^
**Dominio 1 - Objetivos**	0,802
Incluye el tema propuesto	0,820
Es adecuado para el proceso de enseñanza-aprendizaje	0,900
Aclara las dudas sobre el tema	0,562
Propicia la reflexión sobre el tema	0,850
Fomenta el cambio de comportamiento	0,833
**Dominio 2 - Estructura/** **Presentación**	0,816
Lenguaje adecuado al público objetivo	0,930
Lenguaje apropiado para el material educativo	0,904
Lenguaje interactivo, que permite la participación activa en el proceso educativo	0,833
Información correcta	0,625
Información objetiva	0,850
Información clara	0,625
Información necesaria	0,722
Secuencia lógica	0,820
Tema actual	0,878
Tamaño de texto adecuado	0,878
**Dominio 3 - Relevancia**	0,953
Estimula el aprendizaje	0,954
Contribuye al conocimiento en el área	0,954
Despierta el interés por el tema	0,952
**IVC** ^†^ **Total**	0,838

*IVCES = Instrumento de Validación de Contenidos Educativos en Salud; ^†^IVC = Índice de Validez de Contenido

En el campo de la disertación, los expertos (E2, E18 y E21) sugirieron detallar la atención centrada en el niño y la familia, e incluir a los familiares como participantes activos junto con el paciente, para que el procedimiento sea menos traumático. También mencionaron incluir el juguete terapéutico como forma de facilitar la colaboración del paciente durante la inserción de la sonda (E2, E18 y E21).

Tres expertos (E1, E13 y E17) propusieron abordar que el cateterismo gástrico/enteral es una función exclusiva del enfermero, al considerar el equipo de Enfermería, y aclarar cuales son las competencias del enfermero para realizar este procedimiento. Cinco expertos (E1, E4, E14, E16 y E22) mencionaron las especificidades del procedimiento para pacientes neonatales, y señalaron que era necesario detallar esos aspectos en el material o excluir a ese público. Dos expertos (E4 y E17) sugirieron sustituir el término “catéter” por “sonda”, debido a que el primer término se asocia con la terapia intravenosa.

Dos expertos (E19 y E20) mencionaron que se debe prestar atención al marcado y la fijación de la sonda, dado que si se la marca sólo con el bolígrafo se borra, y que la cinta debe estar bien fijada, para evitar que el niño se la trague. En cuanto a la fijación, debe seguir la norma institucional, independientemente de si es en la mejilla o la nariz (E19 y E20). Otra recomendación fue incluir el uso de una placa hidrocoloide para proteger la piel del niño de la fijación de la sonda (E8, E17, E19 y E22).

Respecto a las pruebas de posicionamiento del catéter, algunos enfermeros (E2, E15 y E20) indicaron que existen referencias que contraindican la prueba de auscultación con el estetoscopio, y que el estándar de oro es realizar una radiografía. Sobre el volumen de aire inyectado para la prueba de auscultación, tres expertos (E1, E18 y E19) destacaron que era necesario describir que es diferente y varía según la franja etaria. Respecto a la retirada del alambre guía o mandril del catéter, se señaló que se realiza después de la inserción del catéter, y antes de que el paciente vaya a radiografía (E2, E13, E18 y E20).

Las contribuciones fueron positivas, los expertos evaluaron el material como “muy didáctico” (E2) y “bien diseñado” (E22). Otros expertos felicitaron a los autores por la elección del tema (E15), y consideraron que “la temática, el contenido y el formato del guion eran relevantes” (E13). El guion también fue caracterizado como “extraordinario y de excelente calidad” (E21).

Luego de que el comité de expertos realizara la evaluación y validación, se actualizó el guion en función de sus consideraciones. El video fue grabado en el Laboratorio de Habilidades de la UFCSPA, la versión inicialmente duraba 10 minutos y 1 segundo y se denominada: “Cateterismo gástrico/enteral en Pediatría” (*Sondagem gástrica/enteral em Pediatria*).

El video tiene escenas grabadas con un maniquí de simulación pediátrica, imágenes de los materiales utilizados en el procedimiento y pantallas con contenido teórico. Su contenido abarca desde la preparación de materiales, hasta técnicas específicas de inserción y verificación del correcto posicionamiento de la sonda, y cuenta con diferentes recursos visuales, en forma de pantallas, que le brindan base teórica y legal. En la Tabla 2 se muestra la evaluación de los expertos de los 13 ítems que componen el Instrumento de Validación del Video.

**Tabla 2 -:** Validación del video, según el Instrumento de Validación del Video (n = 12). Porto Alegre, RS, Brasil, 2022-2023

**Variables**	IVC*
**Dominio 1 – Funcionalidad**	0,909
El video presenta de forma clara y adecuada los aspectos importantes del procedimiento	0,909
El video facilita el proceso de enseñanza-aprendizaje del procedimiento	0,909
**Dominio 2 - Usabilidad**	0,896
El video permite aprender fácilmente los conceptos teóricos utilizados y la técnica del procedimiento	0,857
El video les permite a los profesionales de enfermería replicar la técnica correcta del procedimiento en la práctica profesional	0,857
Secuencia lógica	0,956
Tamaño de texto adecuado	0,909
**Dominio 3 – Eficacia**	0,933
La duración del video es adecuada para aprender el contenido	0,909
El video es atractivo/mantiene la atención del espectador.	0,956
**Dominio 4 – Técnica Audiovisual**	0,985
La calidad de la imagen de video es adecuada para observar las escenas	0,956
El tono y la voz de los narradores son claros y apropiados	1,000
El vocabulario utilizado en el video es eficiente y comprensible para el público objetivo	1,000
**Dominio 5- Ambiente**	1,000
El entorno utilizado para grabar las escenas se considera adecuado	1,000
El material utilizado para realizar el procedimiento es correcto	1,000
IVC* Total	0,942

*IVC = Índice de Validez de Contenido

En el campo descriptivo, los expertos (E1; E5; E7; E17 y E19) hicieron sugerencias sobre el momento de retirar la guía y la forma de fijar y medir la sonda en el video. Además, se recomendó incluir que es el médico quien debe indicar la retirada de la sonda para nutrición enteral (E17).

Con respecto a las pruebas de posicionamiento de la sonda, se sugirió brindar mayor detalle sobre la técnica de auscultación de la región epigástrica (E22); señalan que la prueba de pH tiene baja precisión porque se ve afectada por las sustancias con las que entra en contacto el jugo gástrico (E17); y que, si bien, los rayos X son el estándar de oro, deben usarse con precaución (E5). También se destacó que hay que utilizar más de una técnica para confirmar el posicionamiento correcto de la sonda (E5 y E21).

Un experto (E17) mencionó que el calibre de sonda utilizado para los niños puede ser a partir de 6 French, y que al momento de elegir el calibre de la sonda hay que considerar las necesidades del niño que el dispositivo debe satisfacer (E5). Se recomendó enumerar los aspectos que diferencian las sondas gástricas y enterales, los tipos de sondas disponibles, las contraindicaciones para la realización del procedimiento y la orientación que se debe dar a la familia sobre el procedimiento (E17 y E22).

En cuanto a la técnica audiovisual, los expertos E7 y E19 señalaron que algunas pantallas informativas tenían corto tiempo de visualización. Dieron orientación sobre cómo narrar toda la información del material (E7); distribuir en más pantallas la información contenida en cada una de ellas (E1), y mostrar el momento de fijar la sonda y retirar el mandril desde un ángulo más cercano (E19 y E21).

Las sugerencias realizadas por los expertos fueron analizadas y comparadas con la literatura para luego incluirlas. El video ha sido actualizado y la versión final dura 10’10”. Posteriormente, el video se subió al canal de *YouTube* ^®^ del Grupo de Investigación, a través del *link* https://bit.ly/47Suc7l.

## Discusión

El predominio de enfermeras de edad adulta media en los expertos refleja los datos de una investigación sobre la profesión en Brasil, en la que el 86,2% son mujeres y el 66,6% tienen más de 40 años^(16)^. La muestra incluyó expertos de tres de las cinco regiones de Brasil, no hubo evaluadores de las regiones Centro-Oeste y Norte. Esto coincide con los datos de investigaciones brasileñas que revelan que hay una menor concentración de enfermeros en esas dos regiones del país, 8,2% y 6,7%, respectivamente^(16)^.

Al analizar la Unidad Federativa de los participantes, el estado de Rio Grande do Sul representó el 39,1% de la muestra. Es probable que un número más significativo de enfermeros en este estado se deba al origen del estudio.

El tiempo de experiencia profesional y la calificación de los expertos son similares a los de otros estudios que validaron materiales educativos, que exigen que tengan mayor título y especialización^(17 - 22)^. La puntuación media de Fehring (adaptada) fue de 11 y osciló entre ocho y 12 puntos. Por lo tanto, estos resultados demuestran que el perfil de los participantes de este estudio es distinguido, lo que valida la experiencia del comité.

En la elaboración del guion/video se buscó presentar contenido teórico relacionado con el procedimiento de cateterismo gástrico/enteral, tales como: concepto, indicaciones/contraindicaciones, objetivo, marco legal, materiales necesarios y técnicas lúdicas. Además, también se abordaron algunos puntos controvertidos en la realización del procedimiento, como medición de la longitud y diámetro de la sonda, posición/sujeción del niño, estabilización del dispositivo y pruebas de confirmación de posicionamiento.

En los últimos cinco años hubo un aumento de la producción de videos educativos en la enseñanza de Enfermería, impulsado por la explosión digital, las recientes demandas de la enseñanza y los cambios en los perfiles de los estudiantes, que favorecieron la inserción de nuevas herramientas educativas. Una revisión integradora, publicada en 2023, encontró 19 artículos sobre producción de videos en Enfermería, la mayoría utilizó las fases de preproducción, producción, postproducción y validación por expertos, al igual que en esta investigación^(9)^.

Este método de elaboración ha mostrado resultados prometedores cuando se analizó el resultado del video en la población objetivo. Un ensayo clínico aleatorizado (ECA), cuyo objetivo era evaluar la efectividad de un video educativo sobre el conocimiento de 100 pacientes hospitalizados sobre prácticas seguras en el período perioperatorio, identificó que el grupo intervención (video educativo) registró un aumento significativamente mayor del conocimiento (t =3,72 ±1,84; p<0,001) que el grupo control (orientaciones estándar)^(23)^.

Otro estudio demostró que los estudiantes de enfermería de habla portuguesa adquirieron mayores conocimientos sobre la punción venosa periférica después de utilizar un video educativo validado sobre el tema^(24)^. Por lo tanto, la propuesta de creación y validación de videos educativos sobre situaciones de atención de enfermería puede favorecer el desarrollo del conocimiento y de las habilidades de los profesionales^(25 - 26)^, como se propone en este estudio. Cuando el material no es evaluado por expertos, puede tener errores de contenido y un diseño poco atractivo, lo que puede desmotivar el uso por parte del público objetivo y fomentar prácticas inapropiadas^(27)^.

El guion del video sobre cateterismo gástrico/enteral en niños alcanzó un IVC total de 0,838 y el video un IVC total de 0,942, y cada dominio osciló entre 0,8002 y 0,952 (guion) y entre 0,896 y 1,0 (video). Por lo tanto, ambos productos se consideran adecuados^(15)^. Otros estudios que validaron videos educativos, con diferentes temáticas, poblaciones e instrumentos, también utilizaron como punto de corte 0,8 para el IVC^(23 , 25 , 28 - 30)^.

Un estudio metodológico que creó y validó un video educativo para estudiantes de Enfermería sobre paro cardiorrespiratorio obstétrico obtuvo un IVC total de 0,99, y los dominios IVCES oscilaron entre 0,95 y 1^(28)^. Otra investigación, que utilizó el mismo instrumento, validó siete videos educativos para promover la seguridad y la salud en el trabajo de profesionales que se desempeñan en la Atención Primaria de Salud, y el dominio total osciló entre 0,88 y 0,96. En IVC del dominio “objetivos”, osciló entre 0,84 y 0,95; el de “estructura/presentación” entre 0,91 y 0,99; y el de “relevancia” entre 0,81 y 0,95^(25)^, estos resultados son similares a los del presente estudio.

La validación del guion aumentó la calidad del contenido para la posterior creación del video, que presentó un IVC superior al del guion. Los aportes de los expertos que evaluaron el guion y el video fueron fundamentales para mejorar la información y garantizar la calidad de los productos elaborados, especialmente del contenido controversial, como la medición de la longitud de la sonda y las pruebas confirmatorias de posicionamiento. Como las sugerencias fueron puntuales, debido a que las preguntas de los instrumentos eran abiertas, y todos los valores del IVC de los dominios de los dos instrumentos fueron superiores al punto de corte establecido (0,80), se realizó sólo una ronda de evaluación y los cambios se realizaron en función del análisis de los comentarios, la comparación con la literatura^(31 - 34)^ y la decisión final de los autores.

En la validación del guion del video se realizaron observaciones sobre los objetivos del procedimiento y la necesidad de incluir resoluciones del Consejo Federal de Enfermería (COFEN) en el procedimiento de cateterismo gástrico/enteral y contención mecánica del paciente, que fueron agregadas. Los expertos también señalaron que era necesario enfatizar la atención centrada en el niño y la familia, para que el ingreso hospitalario y los procedimientos realizados durante este período sean menos traumáticos. Para brindar una atención integral al paciente pediátrico, se le debe explicar claramente el procedimiento al niño, según su etapa de desarrollo, y a los familiares, e incluirlos en la atención. Una forma de hacerlo es mediante el uso del juguete terapéutico, instrumento atractivo para el niño, que permite demostrar de forma lúdica lo que se realizará, y permite que el procedimiento sea más tranquilo y humanizado^(35)^.

En el guion elaborado en un principio, no se distinguía el procedimiento en función de las diferentes franjas etarias en Pediatría. Por ello, los expertos destacaron que era importante incluir las especificidades del período neonatal. Dicho periodo abarca desde el nacimiento hasta el día 28 de vida, y es una fase reducida^(36)^. Por lo tanto, al comprobar las particularidades de esa fase del desarrollo, se decidió no incluir a ese público en el material, ya que la mayor parte del público objetivo son niños con más de 28 días de vida. Esta decisión fue informada en el guion final y en el video.

Algunos expertos mencionaron que era necesario cambiar el término catéter, inicialmente utilizado para elaborar el guion, por el término sonda, tradicional e históricamente utilizado en los servicios de salud y en el ámbito universitario. Se decidió cambiar el término “catéter” por “sonda”, porque es el más utilizado en el país, aunque técnicamente es adecuado usar “catéter” o “tubo”, y este último es el más utilizado en las publicaciones en inglés^(1 - 2 , 5 - 7 , 31 - 33)^.

Después de realizar el procedimiento de cateterismo gástrico/enteral es necesario tomar algunas precauciones, como asegurarse de que la ubicación del dispositivo en el tracto gastrointestinal sea la adecuada. Al respecto, los expertos informaron que las mejores prácticas ya no indicaban la realización de una prueba de auscultación de la región epigástrica, sino que era necesario realizar una radiografía. Algunos estudios no recomiendan y desaconsejan el uso de la prueba de auscultación debido a que se pueden auscultar ruidos en la región epigástrica, independientemente de si la punta de la sonda está ubicada en el estómago, el esófago o el tracto respiratorio^(31)^. Siguiendo los aportes de los expertos y los hallazgos de la literatura^(31 - 33)^, se destacaron los dos aspectos en el guion cuando se abordan las pruebas para confirmar el posicionamiento de la sonda.

En la literatura hay divergencias sobre la precisión de los métodos. Por eso, el video destacó que es importante realizar siempre dos técnicas al lado de la cama para confirmar el correcto posicionamiento de la sonda antes de usarla^(31 - 33)^. La radiografía es considerada el estándar de oro para confirmar el posicionamiento de la sonda, pero, como menciona el evaluador y fue incorporado al video, debe usarse con precaución, ya que la exposición repetitiva a la radiación puede ser peligrosa para el niño^(31 - 33)^.

Un experto cuestionó la precisión de la prueba de pH, dado que el pH puede verse afectado por la administración reciente de dieta y el uso de medicamentos que inhiben la secreción gástrica. La literatura demuestra que no hay diferencia significativa entre los pacientes que recibieron el medicamento o alimento y los que no, pero esto puede confundir a los profesionales, por lo que es importante utilizar una segunda técnica para confirmar la posición^(31 - 33)^. Por lo tanto, se mantuvo la recomendación de la prueba de pH en el video, debido a que se puede considerar una prueba sensible para identificar la posición de la sonda^(31 - 33)^.

Un experto señaló que era necesario describir mejor la prueba de auscultación de la región epigástrica. Esta técnica estaba descrita en el guion del video, pero no había sido incorporada al video en formato de texto, porque había sido incluida en la filmación. Para aclarar la información, se agregó en el video una descripción de la técnica.

Otro cuidado importante para garantizar el confort del paciente es el de la piel y las mucosas. Para ello, algunos expertos destacaron que era importante utilizar placas de hidrocoloide para prefijar la sonda. Esa sugerencia fue incorporada en las correcciones del guion, dado que se trata de una tecnología que reduce la humedad, la fricción y el corte, y se indica para prevenir lesiones por presión, incluidas las relacionadas con el uso de dispositivos médicos^(37)^.

Sin embargo, hay que aclarar que, como es un material costoso, no siempre será fácil acceder a él en los diferentes niveles de atención y/o servicios, especialmente si se consideran las diferencias que presentan los servicios de salud en Brasil.

Al validar el guion y, posteriormente, el video, los expertos abordaron las diferentes formas de fijar/estabilizar la sonda, es decir, utilizando cuerda o estabilizándola en el labio superior, mentón o región temporomandibular. Debido a que aportaron diferentes orientaciones se mantuvo lo que ya estaba en el guion inicial, dado que no existe un estándar de oro para ello y hay que considerar las particularidades del paciente y seguir las pautas de la institución de salud, siempre valorando la seguridad y comodidad del paciente para garantizar el cuidado de la piel. En el guion se incluyó una observación sobre la fijación de la sonda cuando se inserta por vía oral, que no había sido especificada previamente. En este estudio, se consideró que la sonda debía fijarse, mediante cinta adhesiva microporosa, a la mejilla del niño y a la nariz, si se coloca a través de las fosas nasales^(38)^. Cuando se inserta por vía oral, hay que posicionar la sonda centralmente y estabilizarla sobre el labio superior cuando sea posible^(39)^.

Todavía existen divergencias en el conocimiento sobre cómo insertar y mantener la sonda gástrica/enteral, y hay algunas lagunas en la literatura. Se considera que es necesario aplicar rigor técnico-científico y garantizar la seguridad del paciente en todas las etapas del procedimiento. Un ejemplo de esta divergencia es el momento de retirar la guía, mencionado por los expertos que evaluaron el guion y el video, que dieron diferentes recomendaciones. Algunos autores recomiendan retirarla después de realizar la radiografía^(38)^, otros, a su vez, aconsejan que se puede retirar inmediatamente después de insertar el catéter y estabilizar el dispositivo en la piel, lo que puede favorecer la migración de la punta de la sonda y su posicionamiento final incluso antes de realizar el examen de imagen^(40)^. En este estudio se decidió adoptar la recomendación de retirar la guía después de realizar las pruebas de posicionamiento y antes de enviar al paciente a radiografía^(34 , 40)^.

En la validación del video, se recomendó revisar la forma de medir la longitud a insertar de la sonda. En la literatura se describen varios enfoques, como la medición basada en los aspectos morfológicos NEX (*Nose*, *Earlobe*, *Xiphoid*) y NEMU (*Nose*, *Earlobe*, *Mid-Umbilicus*) para el cateterismo gástrico y el método NEX, con cuatro centímetros más para el cateterismo enteral^(31 , 33)^. Además, los expertos destacaron que se podía realizar una medición basada en la relación entre edad y altura. Se decidió mantener la forma utilizada en el video, y medir la posición gástrica desde la punta de la nariz/borde de la boca hasta el lóbulo de la oreja, y de allí hasta el espacio medio entre la apófisis xifoides y la cicatriz umbilical; y la posición entérica desde la punta de la nariz hasta el lóbulo de la oreja, de allí a la apófisis xifoides y luego a la cicatriz umbilical^(34)^.

Los expertos también hicieron sugerencias sobre el calibre de la sonda, que se implementaron en el video. La primera se refiere a que se pueden utilizar sondas a partir de 6 French en niños, como indica la literatura^(34)^, y en el video se recomendó a partir de 6 French^(36)^. Cabe señalar que, a la hora de elegir el calibre de la sonda, se debe optar por la de menor diámetro, considerando las características del niño y las necesidades terapéuticas que debe cubrir el dispositivo.

No se agregó cierta información sugerida, como: contraindicaciones para la realización del procedimiento y los aspectos que diferencian las sondas gástricas y enterales, para evitar que el video fuera largo. De todas formas, se encuentra disponible como material complementario al video y se puede acceder a él a través del *Quick Response Code* y/o en la descripción del video en *YouTube* ^®^. En cuanto al tiempo de duración, 10 minutos y 10 segundos, se cree que afecta la participación y el aprendizaje del espectador, por ello, se respetaron las recomendaciones que estipulan que debe durar de seis a 15 minutos^(41 - 42)^, al igual que otros estudios que validaron videos educativos para Enfermería^(28 - 30 , 43 - 44)^.

En cuanto a la técnica audiovisual, los autores del video optaron por aumentar el tiempo de visualización de algunas pantallas informativas, para permitir la lectura sin necesidad de pausar el video. Además, se cambió el ángulo de grabación para fijar la sonda y retirar el mandril, según lo recomendado. Sin embargo, no se aceptó la sugerencia sobre la narración de toda la información contenida en el material.

El guion y el video, elaborados con rigor científico y validados por expertos, pueden ser utilizados como estrategia para diversificar los procesos de enseñanza-aprendizaje de enfermeros y estudiantes de enfermería, en el aula, en el laboratorio o en actividades complementarias. El video, titulado: Cateterismo gástrico/enteral en Pediatría, realizado a partir del guion, lo puede utilizar el profesional/estudiante al momento de realizar el procedimiento, es de fácil consulta, sobre todo porque está disponible en plataformas de libre acceso en Internet, como *YouTube* ^®^, y se puede acceder al guion a través del *link* provisto en la descripción del video, al igual que al material que se utilizó para la elaboración.

Los resultados y el producto (video) son originales e inéditos, dado que, hasta donde se sabe, no hay ninguna publicación sobre un estudio para crear un video educativo orientado al público objetivo y sobre el tema de la presente investigación. Por lo tanto, se destaca la originalidad y el potencial que tienen para ayudar a los profesionales a desarrollar habilidades teórico-práctico en el tema de procedimientos de cateterismo gástrico y enteral en niños.

Se considera una limitación que haya una mayor participación local-regional de los expertos que realizaron la validación de los productos, esto puede limitar el uso de los productos en las regiones Centro-Oeste y Norte de Brasil, debido a las diferentes culturas en el país. La pérdida de 11 expertos en la última etapa no invalida los resultados, ya que los 12 video evaluadores superaron el mínimo estipulado en la literatura, que es seis^(15)^. Al no encontrarse otras investigaciones que incluyan el tema y el público objetivo (lo que destaca su originalidad), existe una restricción para comparar los datos con otros estudios. Por último, el uso de IVC, fundamental en el proceso de desarrollo de productos, tiene como limitación la subjetividad de los expertos^(15)^, que se trató de minimizar al tomar las decisiones basándose en la literatura.

## Conclusión

El video “Cateterismo gástrico/enteral en Pediatría” fue validado por el comité de expertos. El estudio contribuye al avance del conocimiento científico y la práctica de los cuidados de enfermería al brindar un recurso educativo con contenido elaborado con rigor científico, de fácil acceso y gratuito.

El producto puede ser utilizado de forma independiente en diferentes propuestas pedagógicas, siempre y cuando se cumpla con la licencia *Creative Commons* Atribución 4.0 Internacional. Se sugiere realizar más estudios para analizar el efecto sobre el conocimiento y las habilidades de enfermeros y estudiantes.
